# Structural basis of HIV-1 maturation inhibitor binding and activity

**DOI:** 10.1038/s41467-023-36569-y

**Published:** 2023-03-04

**Authors:** Sucharita Sarkar, Kaneil K. Zadrozny, Roman Zadorozhnyi, Ryan W. Russell, Caitlin M. Quinn, Alex Kleinpeter, Sherimay Ablan, Hamed Meshkin, Juan R. Perilla, Eric O. Freed, Barbie K. Ganser-Pornillos, Owen Pornillos, Angela M. Gronenborn, Tatyana Polenova

**Affiliations:** 1 Department of Chemistry and Biochemistry, University of Delaware, Newark, DE 19716 USA; 2 Pittsburgh Center for HIV Protein Interactions, University of Pittsburgh School of Medicine, 1051 Biomedical Science Tower 3, 3501 Fifth Ave., Pittsburgh, PA 15261 USA; 3 Department of Molecular Physiology and Biological Physics, University of Virginia School of Medicine, Charlottesville, VA 22908 USA; 4 HIV Dynamics and Replication Program, Center for Cancer Research, National Cancer Institute, Frederick, MD 21702-1201 USA; 5 Department of Structural Biology, University of Pittsburgh School of Medicine, 3501 Fifth Ave., Pittsburgh, PA 15261 USA

**Keywords:** Solid-state NMR, Supramolecular assembly, Viral proteins, Mechanism of action, Virus structures

## Abstract

HIV-1 maturation inhibitors (MIs), Bevirimat (BVM) and its analogs interfere with the catalytic cleavage of spacer peptide 1 (SP1) from the capsid protein C-terminal domain (CA_CTD_), by binding to and stabilizing the CA_CTD_-SP1 region. MIs are under development as alternative drugs to augment current antiretroviral therapies. Although promising, their mechanism of action and associated virus resistance pathways remain poorly understood at the molecular, biochemical, and structural levels. We report atomic-resolution magic-angle-spinning NMR structures of microcrystalline assemblies of CA_CTD_-SP1 complexed with BVM and/or the assembly cofactor inositol hexakisphosphate (IP6). Our results reveal a mechanism by which BVM disrupts maturation, tightening the 6-helix bundle pore and quenching the motions of SP1 and the simultaneously bound IP6. In addition, BVM-resistant SP1-A1V and SP1-V7A variants exhibit distinct conformational and binding characteristics. Taken together, our study provides a structural explanation for BVM resistance as well as guidance for the design of new MIs.

## Abstract (web summary)

HIV maturation inhibitors such as bevirimat (BVM) interfering with Gag processing are emerging as alternative anti-retroviral drug candidates. Here, the authors report structures of assemblies of HIV-1 Gag fragments spanning the CA C-terminal domain and SP1 region bound to BVM.

## Introduction

HIV-1 maturation is triggered by the viral protease that cleaves the structural Gag polyprotein precursor into its constituent domains^1–3^. HIV-1 Gag harbors five proteolytic cleavage sites between its four major structural and functional domains and two spacer peptides: MA, CA, SP1, NC, SP2, and p6. Processing proceeds at different rates, with the SP1-NC site cleaved the fastest and the CA-SP1 site last^4^. Genetic and enzymatic studies showed that inhibition of cleavage or even slowing cleavage at the CA-SP1 site is sufficient to significantly disrupt the maturation process and abrogate virus infectivity. Indeed, maturation inhibitors (MIs) that interfere with CA-SP1 processing are emerging as attractive candidates for augmenting the current arsenal of treatments for HIV-infection^5–9^.

Biochemical and structural studies revealed that slow cleavage of CA-SP1 is due to structural sequestration of the proteolysis site^10–13^. Within the assembled immature HIV-1 Gag lattice, the CA-SP1 junction folds into an α-helix (the junction helix), which self-associates into a 6-helix bundle, stabilizing the Gag hexamer^13,14^. The scissile bond between CA-L231 and SP1-A1 is located in the middle of the junction helix and is occluded inside the 6-helix bundle. Therefore, for the protease to gain access to this site, the 6-helix bundle must at least partially unfold. Although the detailed mechanism of inhibition has not been ascertained, small-molecule MIs, such as 3-*O*-(3’,3’-dimethylsuccinyl)-betulinic acid (Bevirimat or BVM), 1-[2-(4-tert-butylphenyl)-2-(2,3-dihydro-1H-inden-2-ylamino)ethyl]-3-(trifluoromethyl)pyridin-2-one (PF-46396) and their analogs are thought to interfere with proteolysis by binding to the CA-SP1 junction and stabilizing the 6-helix bundle^6,7,15–18^. Thus, MIs do not directly interfere with substrate binding but rather act indirectly by inhibiting the unfolding of the 6-helix bundle and in effect impeding access of the protease to its substrate.

Despite being potent inhibitors of HIV infection in laboratory settings, MIs have not yet been approved for clinical use. BVM underwent phase I and phase II clinical trials, during which significant, dose-dependent viral load reductions in HIV-1-infected individuals were observed^19^. However, further studies revealed that in up to 50% of patients, BVM did not affect viral loads^20,21^. This BVM resistance is associated with naturally occurring viral sequence polymorphs, in particular SP1 amino acid changes at residues 7 and 8 (SP1-V7A, -V7M, -T8Δ and -T8N)^20^. In addition, BVM resistant variants were generated through multiple rounds of selection against BVM in vitro, resulting in amino acid changes in SP1 residues 1 and 3 (SP1-A1V, -A3T, and -A3V); of these, SP1-A1V does not impair viral replication^10^.

Inositol hexakisphosphate (IP6), a negatively charged small molecule that is abundant in cells, also stabilizes the CA-SP1 junction by binding to the 6-helix bundle. In contrast to BVM, which binds in the center of the helical bundle^14,18^, IP6 is located just above the 6-helix bundle and forms salt bridges with two rings of lysine side chains (CA-K158 and CA-K227)^22^. Although BVM also contains negatively charged carboxylates, it has not been established whether these can compete with IP6 for interacting with the lysine rings.

To assess how MIs bind to the CA-SP1 site and elucidate the mechanisms that underlie BVM resistance, we determined magic angle spinning (MAS) NMR atomic-resolution structures of microcrystalline complexes of a HIV-1 Gag fragment spanning the CA C-terminal domain (CA_CTD_) and SP1 regions (CA_CTD_-SP1), in the presence of BVM and/or IP6. Structures were calculated based on a large number of distance restraints, which were derived from carbon-carbon and carbon-proton correlations in high-quality spectra. Intermolecular correlations between ligand and protein resonances allowed us to verify simultaneous binding of BVM and IP6, and to unambiguously assign the binding orientation of one BVM molecule inside the CA-SP1 junction 6-helix bundle. Overall, the structures reported herein provide unprecedented atomic-level details of how BVM and IP6 interact with CA_CTD_-SP1, unavailable from any other structural techniques, and explain the structural basis of BVM-mediated maturation inhibition and resistance of SP1-A1V and SP1-V7A variants. Our study also highlights the power of MAS NMR spectroscopy for directly observing and structurally characterizing bound small molecules in large macromolecular assemblies with atomic-level detail.

## Results

### Resonance assignments and distance restraints

**Fig. 1:**
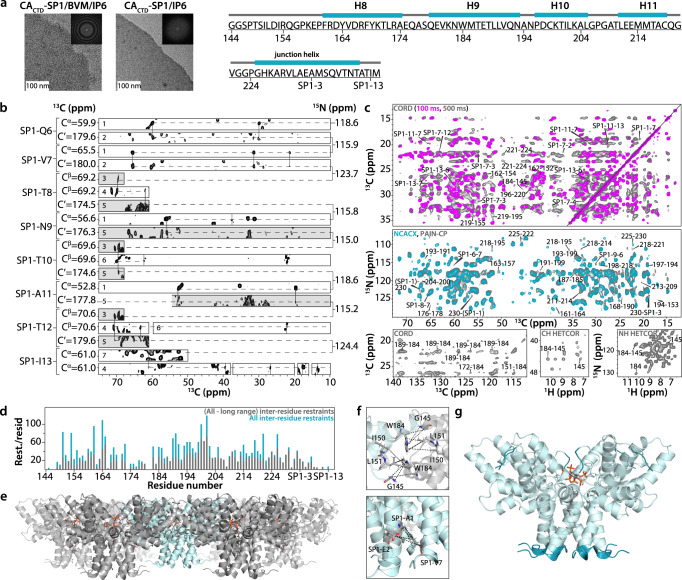
MAS NMR spectra and structure of CA_CTD_-SP1 crystalline array. **a** Negative stain TEM images of CA_CTD_-SP1 microcrystals assembled with IP6, in the presence (left) or absence of BVM (middle). Insets show the computed Fourier transforms of the images, indicating the expected hexagonal lattices and unit cell spacings. The scale bars are 100 nm. Amino acid sequence of CA_CTD_-SP1 (right). **b** Representative strips of 3D and 2D MAS NMR spectra of U-^13^C,^15^N-CA_CTD_-SP1/BVM/SO_4_ (white strips) and U-^13^C,^15^N-CA_CTD_-SP1/IP6 (gray strips) crystalline arrays, illustrating sequential assignments for SP1 residues Q6-I13. The MAS NMR spectra are labeled as follows: 3D NCACX (1), 3D NCOCX (2), 2D NCACX at −79 °C (3), 2D INADEQUATE (4), 2D NCOCX at −79 °C (5), 2D CORD (6), 2D NCACX (7). No significant chemical shift perturbations were detected for the free vs. BVM- or IP6-bound samples (see Supplementary Fig. 3). **c** Top panel: Superposition of selected regions of 2D CORD spectra of U-^13^C,^15^N-CA_CTD_-SP1/BVM/IP6 crystalline arrays for different mixing times: 100 ms (magenta) and 500 ms (gray). Middle panel: Superposition of selected regions of 2D NCACX (cyan) and 2D PAIN-CP (gray) spectra of U-^13^C,^15^N-CA_CTD_-SP1/BVM/IP6 crystalline arrays. Unambiguous long-range and inter-residue correlations of SP1 residues are labeled by amino acid number in the sequence. Bottom panel: Inter-hexamer correlations are shown for the selected regions of 2D CORD (left) and CH HETCOR (middle) spectra of U-^13^C,^15^N-CA_CTD_-SP1/BVM/IP6, and (H)NH HETCOR (right) spectra of U-^13^C,^15^N,^2^H-CA_CTD_-SP1/IP6 (Buffer B). **d** Number of long-range and all inter-residue MAS NMR restraints per residue plotted against the residue number. **e** Side view of hexamer of hexamers of BVM- and IP6-bound CA_CTD_-SP1 arrays. **f** Expansion of inter-hexamer (top panel) and inter-chain (bottom panel) regions showing distances obtained from MAS NMR correlation experiments. **g** MAS NMR structure of a single hexamer of BVM- and IP6-bound CA_CTD_-SP1 crystalline array. The residues detected by MAS NMR and not modeled in the X-ray and cryo-EM structures^13, 14^ are shown in darker cyan.

Negative-stain transmission electron microscopy (TEM) images confirmed previous findings that CA_CTD_-SP1 formed microcrystalline assemblies in the presence of IP6^22^ and that the assemblies appeared similar in the presence or absence of BVM (Fig. 1a). MAS NMR experiments were conducted using eleven sets of samples, prepared with different combinations of isotopic labels, and in the presence or absence of BVM and/or IP6 (summarized in Supplementary Table 1). A total of fourteen one-dimensional (1D), seventy-one two-dimensional (2D), and six three-dimensional (3D) spectra were recorded. The sensitivity and the resolution of the data sets are exceptionally high and permitted almost complete (96%) backbone resonance assignments. Overall, 8377 cross peaks were assigned (Table 1; all assignments are summarized in Supplementary Fig. 1 and Supplementary Data 1). Importantly, the MAS NMR spectra provide clear ^13^C chemical shift signatures for mature vs. immature lattices (see Supplementary Fig. 2). All CA_CTD_ tail and SP1 residues, except SP1-M14, give rise to distinct, well-resolved peaks in the MAS NMR spectra (shown in Fig. 1b for a stretch of SP1 residues Q6 through I13). Importantly, the resonances of the C-terminal tail residues G144-S146 and SP1 tail residues T8-I13, which are missing in X-ray^13^, microED^18^, and cryo-ET^23^ structures, were directly detected and assigned in the MAS NMR experiments. The orientation of the C-terminal tail at the inter-hexameric interface of CA_CTD_-SP1 crystalline lattice is defined through unambiguous assignment of G145-W184 correlations found in MAS NMR spectra (Fig. 1c). The secondary chemical shifts unequivocally indicate that SP1 residues 1-10 are helical in the absence and presence of BVM. Such helical conformation is fully consistent with the known 6-helix bundle structure of SP1 in the immature Gag lattice^13,14,18,22^, in contrast to the structure in high-salt assembled CA-SP1 without IP6 where SP1 residues are dynamically disordered, as described previously^24^.

**Table 1:** Summary of samples and the number of assigned peaks

ID	Sample	Assigned peak type	No. of assigned peak*
1.	U-^13^C,^15^N-CA_CTD_-SP1/BVM/IP6	Intra-residue	1033
		Sequential (|i-j| = 1)	372
		Medium range (1< |i-j| <5)	718
		Long range (|i-j| ≥5)	735
2.	U-^13^C,^15^N-CA_CTD_-SP1/ IP6	Intra-residue	524
		Sequential (|i-j| = 1)	38
3.	U-^13^C,^15^N-CA_CTD_-SP1/BVM/SO_4_	Intra-residue	776
		Sequential (|i-j| = 1)	394
		Medium range (1< |i-j| <5)	2
		Long range (|i-j| ≥5)	1
4.	U-^13^C,^15^N-CA_CTD_-SP1/SO_4_	Intra-residue	393
5.	U-^13^C,^15^N,^2^H-CA_CTD_-SP1/BVM/IP6	Intra-residue	618
		Sequential (|i-j| = 1)	26
		Medium range (1< |i-j| <5)	3
		Long range (|i-j| ≥5)	14
6.	U-^13^C,^15^N,^2^H-CA_CTD_-SP1/IP6 (Buffer A)	Intra-residue	431
		Sequential (|i-j| = 1)	2
		Long range (|i-j| ≥5)	5
7.	U-^13^C,^15^N,^2^H-CA_CTD_-SP1/IP6 (Buffer B)	Intra-residue	361
		Sequential (|i-j| = 1)	33
		Medium range (1< |i-j| <5)	1
		Long range (|i-j| ≥5)	1
8.	U-^13^C,^15^N,^2^H- CA_CTD_-SP1-V7A/BVM/IP6	Intra-residue	491
		Sequential (|i-j| = 1)	8
		Medium range (1< |i-j| <5)	1
		Long range (|i-j| ≥5)	10
9.	U-^13^C,^15^N,^2^H-CA_CTD_-SP1-V7A/IP6	Intra-residue	457
		Sequential (|i-j| = 1)	8
		Medium range (1< |i-j| <5)	1
		Long range (|i-j| ≥5)	6
10.	U-^13^C,^15^N,^2^H-CA_CTD_-SP1-A1V/BVM/IP6	Intra-residue	445
		Sequential (|i-j| = 1)	6
		Long range (|i-j| ≥5)	6
11.	U-^13^C,^15^N,^2^H-CA_CTD_-SP1-A1V/IP6	Intra-residue	445
		Sequential (|i-j| = 1)	6
		Long range (|i-j| ≥5)	6
*Total assigned peaks*			8377

^*^Cross peaks present in different experiments are counted only once.

A large number of intra-protein correlations were detected in multiple MAS NMR spectra (Fig. 1c), and, given the excellent resolution, no ^13^C isotopically diluted samples were needed to distinguish intramolecular from intermolecular correlations: all cross peaks are well resolved and could be unambiguously assigned, permitting to extract distance restraints. In total, 3048 non-redundant unambiguous protein-protein distance restraints (^13^C-^13^C, ^15^N-^13^C, ^13^C-^1^H, and ^15^N-^1^H) were obtained, comprising 674 medium-range (1< |i-j | <4), 627 long-range (|i-j | ≥5), with 39 long-range inter-chain and 22 long-range inter-hexamer restraints. With nearly 30 non-redundant unambiguous restraints per residue or over 52% C-C restraint completeness (see Table 2) and a very large number of inter-residue restraints (1754) derived from long-range correlations (Fig. 1d), to our knowledge, this study yielded the highest number of distance restraints of any protein MAS NMR investigation to date.

**Table 2:** Summary of MAS NMR restraints and structure statistics

**BVM-Protein restraints**	^**1**^**H(BVM)-**^**13**^**C(protein)**			
Unambiguous	7			
**IP6-protein restraints (For CA**_**CTD**_**-SP1/BVM/IP6)**	^**1**^**H(IP6)-**^**13**^**C(protein)**		^**31**^**P(IP6)-**^**1**^**H(protein)**	
Unambiguous	3		3	
**IP6-protein restraints (For CA**_**CTD**_**-SP1/IP6)**	^**1**^**H(IP6)-**^**13**^**C(protein)**		^**31**^**P(IP6)-**^**1**^**H(protein)**	
Unambiguous	6		0	
**Protein distance restraints**	^**13**^**C-**^**13**^**C**	^**15**^**N-**^**13**^**C**	^**15**^**N-**^**1**^**H**	^**13**^**C-**^**1**^**H**
Unambiguous	2125	537	91	295
Intra-residue	595	358	82	259
Sequential (|i-j| = 1)	227	132	7	26
Medium range (1< |i-j| <5)	641	29	1	3
Long range (|i-j| ≥5)	610	11	0	6
Long range (|i-j| ≥5) (inter-chain)	32	7	0	0
Long range (|i-j| ≥5) (inter-hexamer)	20	0	1	1
Ambiguous	117	4	6	2
Intra-residue	22	1	6	2
Sequential (|i-j| = 1)	13	1	0	0
Medium range (1< |i-j| <5)	34	2	0	0
Long range (|i-j| ≥5)	48	0	0	0
**Total number of unambiguous restraints (protein, BVM, and IP6 restraints)**	3061			
**Restraints/residue**	30			
**Percent completeness**	52% (C-C only)			
**Dihedral angle restraints**				
ϕ	90			
ψ	90			

Structure statistics				
**CA**_**CTD**_**-SP1/BVM/IP6**				
Violations (mean ± s.d.)				
Distance restraints ≥7.2 Å (Å)	0.153 ± 0.002			
Dihedral angle restraints ≥5° (°)	2.579 ± 0.088			
Max. protein-protein distance restraint violation* (Å)	1.892			
Max. protein-ligand distance restraint violation* (Å)	2.787			
Max. dihedral angle restraint violation* (°)	14.295			
Deviations from idealized geometry				
Bond lengths (Å)	0.012 ± 0.000			
Bond angles (°)	0.999 ± 0.014			
Impropers (°)	0.964 ± 0.016			
Average pairwise r.m.s.d. (Å)				
Heavy	1.1 ± 0.1			
Backbone (N, Cα, C)	0.9 ± 0.1			
**CA**_**CTD**_**-SP1/IP6**				
Violations (mean ± s.d.)				
Distance restraints ≥7.2 Å (Å)	0.140 ± 0.001			
Dihedral angle restraints ≥5° (°)	2.646 ± 0.095			
Max. protein-protein distance restraint violation* (Å)	2.444			
Max. protein-ligand distance restraint violation* (Å)	no violations			
Max. dihedral angle restraint violation* (°)	16.020			
Deviations from idealized geometry				
Bond lengths (Å)	0.010 ± 0.000			
Bond angles (°)	0.970 ± 0.006			
Impropers (°)	0.969 ± 0.019			
Average pairwise r.m.s.d. (Å)				
Heavy	1.3 ± 0.2			
Backbone (N, Cα, C)	1.1 ± 0.2			

^*^Pairwise r.m.s. deviations were calculated among 5 lowest energy central hexamers.

**Fig. 2:**
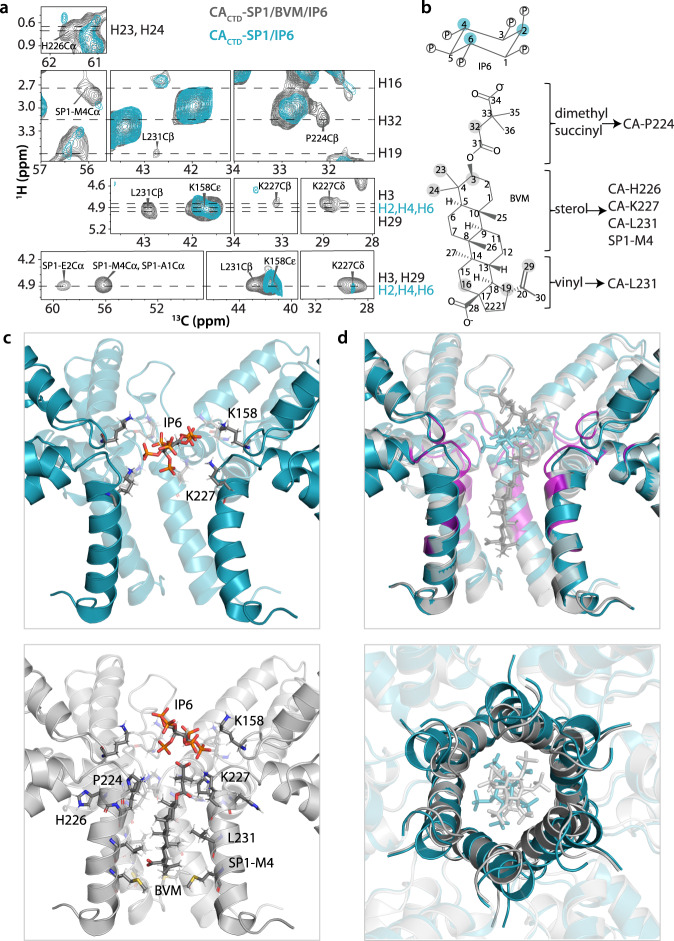
MAS NMR structure of BVM- and IP6-bound CA_CTD_-SP1. **a** Superposition of selected regions of 2D HC CP HETCOR spectra (top three panels) and 2D dREDOR-HETCOR spectra (bottom panel) of U-^13^C,^15^N,^2^H-CA_CTD_-SP1/BVM/IP6 (gray) and U-^13^C,^15^N,^2^H-CA_CTD_-SP1/IP6 (cyan) assemblies. The BVM and IP6 ^1^H atoms and CA_CTD_-SP1 residues with ^13^C atoms are indicated outside and inside each spectrum, respectively. **b** Chemical identity of IP6 and BVM molecules. The IP6 and BVM protons interacting with CA_CTD_-SP1 residues are shown in cyan and gray, respectively. **c** Top panel: IP6 binding mode in the hexamer of CA_CTD_-SP1/IP6 assemblies (dark cyan, PDB: 7R7Q, this work). Bottom panel: IP6 and BVM binding modes in the hexamer of CA_CTD_-SP1/BVM/IP6 assemblies (gray, PDB: 7R7P, this work). Residues interacting with IP6 or BVM are shown as sticks. **d** Superposition of MAS NMR structure of CA_CTD_-SP1/BVM/IP6 and CA_CTD_-SP1/IP6 shown in side view (top) and top view (bottom). BVM binding induces major structural rearrangements of the SP1 helices, resulting in the tightening of the pore and quenching the motions of the simultaneously bound IP6. Residues colored in magenta give rise to high intensity peaks, corresponding to intra- and inter-residue correlations upon BVM binding.

We detected direct correlations between the natural abundance small molecules, IP6 and BVM and the isotopically labelled protein. For IP6, correlations were seen between CA-K158Cε and IP6-H2/H4/H6, indicating that CA-K158 mediates IP6 binding. They were translated into 6 restraints, involving two adjacent chains in the 6-helix bundle for each of the three IP6 protons. Additionally, a very weak correlation was observed with CA-K227Cδ. Correlations with BVM resulted in 7 distance restraints, which unambiguously defined the binding orientation of the inhibitor within the 6-helix bundle. These correlations are summarized in Fig. 2a. A summary of all distance restraints is provided in Table 2 and a plot of all inter-residue contacts is illustrated in Supplementary Fig. 4.

### Higher-order structure of CA_CTD_-SP1 and conformations of BVM and IP6

The structure of a single chain of CA_CTD_-SP1 was calculated using only experimental MAS NMR distance restraints and dihedral restraints. This structure was used to calculate higher order protein structures in complex with BVM and IP6 (details of structure calculation are provided in Materials and Methods section). Higher-order structures (Fig. 1e) were calculated by integrating the experimental MAS NMR restraints (i.e., protein-protein, protein-BVM, and protein-IP6 distance and protein torsion angle restraints) and a hexamer-of-hexamers structural envelope generated from X-ray coordinates of the CA_CTD_-SP1 hexamer (PDB: 5I4T)^13^. The hexamer-of-hexamers unit represents the minimal building block that recapitulates the critical inter-hexamer interfaces in the immature capsid lattice. The MAS NMR-derived CA_CTD_-SP1 inter-chain and inter-hexamer contacts are shown in Fig. 1f.

As described previously^13,14,18,22^, the CA_CTD_-SP1 hexamer exhibits the shape of a goblet, with the globular CA_CTD_ domain forming the cup and the 6-helix bundle CA-SP1 junction fashioning the stem. In the crystal structure of CA_CTD_-SP1 and the cryo-EM structure of full-length Gag, the junction helix terminates around residue SP1-V7, with the remaining residues not visible, likely due to conformational disorder^13,18,22^. Remarkably, the entire SP1 region, including the SP1 tail, except for SP1-M14, is well defined in the MAS NMR structures (Fig. 1g and Supplementary Fig. 5 and 6). MAS NMR chemical shifts predict a helical conformation up to SP1-T10, with the last 4 residues (A11-M14) being in a random coil structure (Fig. 1g). For SP1-E2, S5, T8, N9, T10, T12, and I13 residues, peak intensities are low, suggesting conformational heterogeneity (Fig. 1b), consistent with the X-ray^13^ and cryo-EM data^14^.

In the BVM-bound structure, the inhibitor is located inside the channel formed by the 6-helix bundle, with the sterol ring occupying the hydrophobic interior of the channel and making contacts with protein residues CA-H226, K227, L231, and SP1-M4. The dimethyl succinyl moiety is oriented towards the CA_CTD_ goblet, as unequivocally indicated by H32(BVM)-P224Cβ(protein) and H3(BVM)-K227Cβ(protein) correlations, whereas the vinyl group points towards the SP1 tail, as suggested by H19(BVM)-L231Cβ(protein) and H29(BVM)-L231Cβ(protein) correlations (Fig. 2a, b). Thus, a single BVM-up orientation is observed experimentally, contrary to the conclusions from a recent computational study, where both BVM-up- and BVM-down-orientations were deemed possible.^25^ Moreover, L231 and SP1-M4 interact asymmetrically with the BVM vinyl group, i.e., 3out of 6 chains of the 6-helix bundle exhibit direct contacts with BVM. This insight into the asymmetric interactions of BVM with protein side chains is not available from any of the prior structures.^18,25,26^ Interestingly, a recent computational study suggested that BVM rotates within the CA_CTD_-SP1 hexamer pore on the timescale of the MD simulation and contacts different protomers during this process.^25^ It was also suggested that BVM interacts uniformly with L231 throughout the six helices while preferential interactions are seen with a few SP1-M4 residues. Our experimental results indicate that BVM does not undergo motions inside the six-helix bundle on nano- to slow microsecond timescales and the asymmetry of BVM binding to the protein observed for all interacting residues, including L231 and SP1-M4, has static character. In summary, our structure confirms that BVM organizes the CA-SP1 junction by binding inside the central channel of the 6-helix bundle^14,18^, breaks the symmetry with respect to the side chains and, importantly, defines the orientation of the bound drug.

**Fig. 3:**
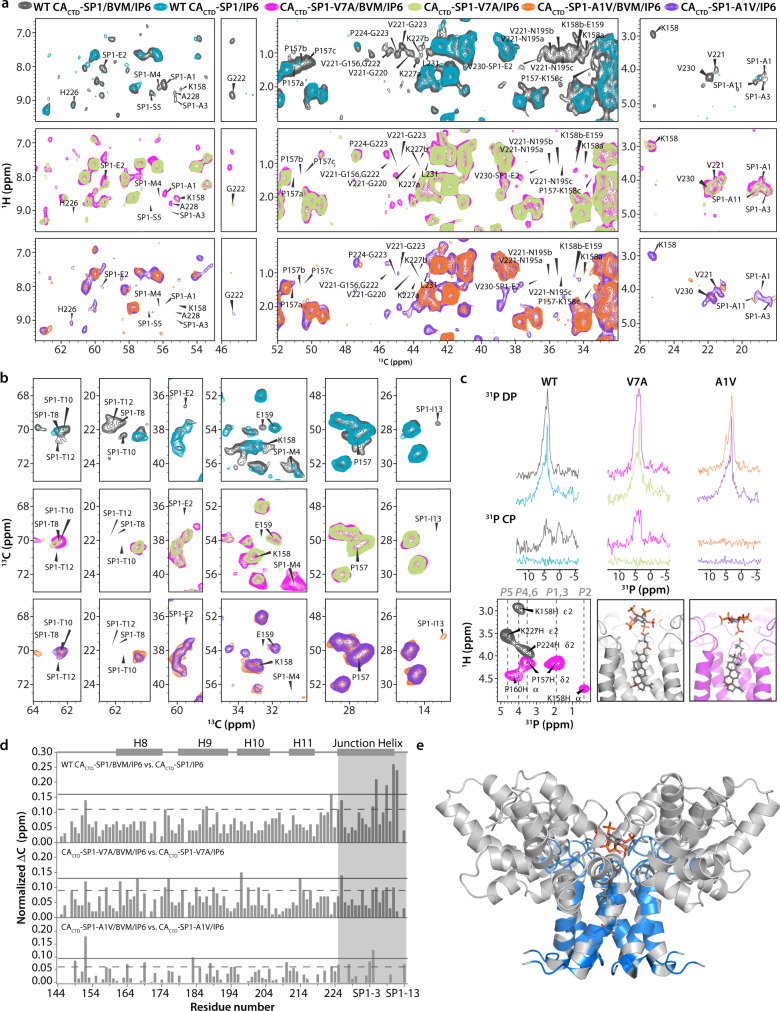
Effect of BVM binding on wild-type (WT) and the SP1-A1V and SP1-V7A variants. **a** Superposition of selected regions of 2D HC CP HETCOR spectra of U-^13^C,^15^N,^2^H-CA_CTD_-SP1/BVM/IP6 (gray) and U-^13^C,^15^N,^2^H-CA_CTD_-SP1/IP6 (cyan) (top panel); U-^13^C,^15^N,^2^H-CA_CTD_-SP1-V7A/BVM/IP6 (magenta) and U-^13^C,^15^N,^2^H-CA_CTD_-SP1-V7A/IP6 (light green) (middle panel); and U-^13^C,^15^N,^2^H-CA_CTD_-SP1-A1V/BVM/IP6 (orange) and U-^13^C,^15^N,^2^H-CA_CTD_-SP1-A1V/IP6 (purple) (bottom panel). Intra- and inter-residue correlations arising upon BVM binding only in WT CA_CTD_-SP1 but not in CA_CTD_-SP1-V7A or CA_CTD_-SP1-A1V are labeled. Residues showing multiple conformers are denoted with a, b, c. **b** Superposition of selected regions of 2D CORD spectra of U-^13^C,^15^N,^2^H-CA_CTD_-SP1/BVM/IP6 (gray) and U-^13^C,^15^N,^2^H-CA_CTD_-SP1/IP6 (cyan) (top panel); U-^13^C,^15^N,^2^H-CA_CTD_-SP1-V7A/BVM/IP6 (magenta) and U-^13^C,^15^N,^2^H-CA_CTD_-SP1-V7A/IP6 (light green) (middle panel); and U-^13^C,^15^N,^2^H-CA_CTD_-SP1-A1V/BVM/IP6 (orange) and U-^13^C,^15^N,^2^H-CA_CTD_-SP1-A1V/IP6 (purple) (bottom panel). Intra-residue cross-peaks exhibiting intensity or chemical shift changes due to BVM binding in WT CA_CTD_-SP1 are labeled. The corresponding correlations are either absent or exhibit small chemical shift perturbations upon BVM binding to CA_CTD_-SP1-V7A/BVM/IP6 and in CA_CTD_-SP1-A1V/BVM/IP6. **c** Top & middle: ^31^P direct polarization (DP, top) and cross polarization (CP, middle) spectra of U-^13^C,^15^N,^2^H-CA_CTD_-SP1/BVM/IP6 (gray), U-^13^C,^15^N,^2^H-CA_CTD_-SP1/IP6 (cyan), U-^13^C,^15^N,^2^H-CA_CTD_-SP1-V7A/BVM/IP6 (magenta), U-^13^C,^15^N,^2^H-CA_CTD_-SP1-V7A/IP6 (light green), U-^13^C,^15^N,^2^H-CA_CTD_-SP1-A1V/BVM/IP6 (orange), and U-^13^C,^15^N,^2^H-CA_CTD_-SP1-A1V/IP6 (purple). Bottom: Overlay of 2D (H)PH HETCOR spectra of U-^13^C,^15^N,^2^H-CA_CTD_-SP1/BVM/IP6 (gray) and U-^13^C,^15^N,^2^H-CA_CTD_-SP1-V7A/BVM/IP6 (magenta) (left). Tilted orientation of IP6 in WT CA_CTD_-SP1/BVM/IP6 (middle) (PDB: 7R7P, this work). Horizontal orientation of IP6 in CA_CTD_-SP1-V7A/BVM/IP6 (right). **d** Chemical shift perturbations induced by BVM binding in WT CA_CTD_-SP1 (top), CA_CTD_-SP1-V7A (middle), and CA_CTD_-SP1-A1V (bottom), plotted against residue number. **e** CA_CTD_-SP1/BVM/IP6 structure with residues exhibiting unique CSPs, intra- and inter-residue correlations, and enhanced peak intensities upon BVM binding to WT CA_CTD_-SP1 shown in blue.

Comparison of the structures in the presence and absence of BVM clearly shows that BVM binding results in apparent tightening of the hexamer pore (Fig. 2c, d) via structural rearrangement in the type-II β-turn and CA-SP1 junction helices. This mechanism of pore tightening discovered herein was not reported in a prior microED study^18^ (Supplementary Fig. 7). Interestingly, in a previously reported solid-state NMR investigation of BVM binding to virus-like particles (VLPs), the possibility of destabilization of the segment preceding the junction helix due to BVM binding was discussed, although not directly supported by experimental data^27^. Those results are consistent with our observation of pore tightening and difference in the position of the type-II β-turn (G220-P224) in the presence of BVM (Fig. 2d). We also observed pronounced conformational heterogeneity of CA-P157, K158, E159, and SP1-M4, which indicates asymmetry of the six protein side chain copies in the hexameric ring. This result provides further evidence that a single, asymmetric BVM molecule is bound inside the 6-helix bundle (Supplementary Fig. 8). Additionally, the SP1 tail becomes less dynamic upon BVM binding, as is evident in the increased intensities of cross peaks of tail residues, together with a concomitant reorientation of side chains of residues close to the binding site, such as CA-P157, K158, E159, N195, G220, V221, G222, G223, P224, K227, V230, L231, and SP1-E2 (Figs. 2d and 3a, top panels, and Supplementary Fig. 9). We suggest that these structural changes all contribute to the stabilizing effect of BVM binding.

Importantly, our data revealed that BVM and IP6 can bind to the CA_CTD_-SP1 hexamer simultaneously, as proposed recently^25,26,28^. This is supported by the observation of distinct sets of correlations between IP6 and CA_CTD_-SP1 in the presence and absence of BVM. Importantly, the mode of interaction between IP6 and CA_CTD_-SP1 is distinct in the presence and absence of BVM. Specifically, the ^1^H-^13^C HETCOR and dREDOR-HETCOR data sets reveal correlations between multiple protons of BVM (H3, H16, H19, H23, H24, H29, H32) and IP6 (H2, H4, H6) with different CA_CTD_-SP1 residues (Fig. 2a), implying that IP6 and BVM binding is not competitive. In the absence of BVM, IP6 binds nearly horizontally inside the neck region of the channel inside the 6-helix bundle, coordinated by six CA-K158 and six CA-K227 residues. This configuration is supported by correlations between the IP6 H2/H4/H6 group of resonances (chemical shifts are too close to be assigned to individual atoms) and the Cε atoms of six CA-K158 side chains (Fig. 2a). A weak H2/H4/H6(IP6) correlation to CA-K227Cδ is observed, confirming a specific contact between IP6 and this residue, consistent with recent reports^22^. Interestingly, no equivalent correlations are observed in the ^1^H-^31^P 1D CPMAS or 2D HETCOR spectra (Fig. 3c), indicating that coordination by the twelve lysine residues is dynamically averaged, with IP6 undergoing local motions inside the pore. In contrast, when BVM is bound, three intense cross peaks appear in the 2D (H)PH HETCOR spectrum of U-^13^C,^15^N,^2^H-CA_CTD_-SP1/BVM/IP6 (Fig. 3c), corresponding to correlations between 3 different phosphorus atoms of IP6 and the side chains of CA-K158, K227, and P224 residues. The presence of intense cross peaks implies that the motion of IP6 in the presence of BVM is arrested. Whether the arrest of IP6 motion is connected to the stabilization the 6-helix bundle is unclear at present, although bound IP6 dynamics can be used to assess MI activity (see below).

### BVM binding to SP1-A1V and SP1-V7A variants

Understanding the mechanisms of resistance to MI inhibitors is of key importance for further development of such molecules as drugs. We therefore evaluated how CA_CTD_-SP1 variants associated with BVM resistance affect binding. CA_CTD_-SP1 assemblies harboring the SP1-A1V and V7A substitutions were prepared and 2D ^1^H-^13^C HETCOR, ^13^C-^13^C CORD, 1D ^31^P DP and CPMAS, and 2D (H)PH HETCOR spectra were recorded in the presence and absence of BVM and/or IP6 (Fig. 3a–c). In the absence of BVM, the peak intensities for most SP1 residues in the A1V and V7A variants are considerably lower than for the wild-type (WT) protein, and many correlations associated with other regions are missing (Supplementary Fig. 10). This result indicates that, in both variants, the SP1 regions are inherently more dynamic than in the WT protein. Comparison of ^13^C chemical shift perturbations (CSPs) induced by BVM for the WT and the two variants, A1V and V7A, revealed most extensive CSPs for the WT assemblies, especially for resonances associated with residues in SP1 (M4, S5, T8, T10, A11), the type-II β-turn (CA-G223, G225) and the junction helix (CA-H226) (Fig. 3d, e). For A1V variant, we found none of the protein-BVM correlations that were observed with WT CA_CTD_-SP1, indicating that BVM binds very weakly, is very mobile or does not bind at all (Supplementary Fig. 11). In contrast, we observed modest, but statistically significant CSPs with V7A, indicating that this variant is bound to BVM more efficiently than A1V (but less efficiently than WT). Interestingly, BVM binding to the V7A assemblies did not render the complex more rigid, in contrast to observations for WT CA_CTD_-SP1, since none of the equivalent correlations are present in the MAS NMR spectra (Fig. 3a, b). Consistent with the CSP data, ^31^P signals of IP6 were observed in 1D ^31^P CPMAS spectra of CA_CTD_-SP1-V7A/BVM/IP6 assemblies, (Fig. 3c), similar to findings for WT CA_CTD_-SP1/BVM/IP6, while they are absent in CA_CTD_-SP1-A1V/BVM/IP6 samples. Therefore, we conclude that IP6 is motionally restricted in the presence of BVM in CA_CTD_-SP1-V7A assemblies, but mobile in assemblies of CA_CTD_-SP1-A1V. Furthermore, IP6 undergoing dynamics in CA_CTD_-SP1-A1V strongly suggests the loss of BVM binding rather than weak binding. Our data correlate well with observations from virology studies^29,30^, revealing that virus-like particles (VLPs) that harbor the SP1-A1V mutation bind BVM less efficiently than WT ^30^. In a prior molecular dynamics study^25^, the loss of symmetry of the 6-helix bundle was also observed. In addition, the CA-SP1 processing is faster in SP1-A1V mutant virions, compared to WT virus, consistent with the notion that the SP1-A1V variant possesses a more conformationally dynamic 6-helix bundle^10,25^. In contrast, CA-SP1 processing in SP1-V7A and WT virions is similar^21^ suggesting different BVM resistance mechanisms for the SP1-V7A and SP1-A1V sequence variants^29^. Taken together, our MAS NMR results suggest that distinct mechanisms can be associated with loss of BVM binding to MI resistant variants.

2D (H)PH HETCOR spectra of WT CA_CTD_-SP1/BVM/IP6 and CA_CTD_-SP1-V7A/BVM/IP6 assemblies reveal distinct ^31^P(IP6)-^1^H(protein) cross peaks (Fig. 3c). For the WT protein, three correlations are present: P4/6(IP6)-K158Hε2(protein), P5(IP6)-K227Hε2(protein), and P4/6(IP6)-P224Hδ2(protein). For CA_CTD_-SP1-V7A, P1/3/4/6(IP6)-P157Hδ2(protein), P2(IP6)-K158Hα(protein), and P4/6(IP6)-P160Hα(protein) correlations are observed (Fig. 3c and Supplementary Fig. 12 and 13). In contrast, no correlations are seen in the CA_CTD_-SP1-A1V spectra, suggesting that IP6 is dynamic. For WT CA_CTD_-SP1, the correlation between a single phosphorus atom (P5) of IP6 and SP1-K227Hε2 suggests that IP6 is facing only one of the six CA-K227 residues. The fact that no correlations to P2 and P1,3 are seen in WT CA_CTD_-SP1 spectra is consistent with a tilted orientation of IP6 (Fig. 3c). For the SP1-V7A variant, the absence of IP6 correlations with CA-K227, together with the presence of IP6 correlations with CA-P157, K158, and P160, suggests that IP6 adopts a horizontal orientation in the neck region (Fig. 3c). We posit that the distinct dynamic properties and orientations adopted by IP6 in the WT, SP1-A1V, and SP1-V7A protein assemblies may correlate with BVM inhibitory activity against the resistant viruses, suggesting the tantalizing possibility that ^31^P MAS NMR of IP6 bound assemblies could be developed for MI screening.

## Discussion

Here, we presented MAS-NMR structures of the CA_CTD_-SP1 lattice in complex with IP6, in the presence or absence of BVM, that reveal atomic-level details of protein-ligand interactions. In particular, our data confirm that BVM binds inside the pore of the CA_CTD_-SP1 6-helix bundle, simultaneously with IP6. Importantly, we unambiguously defined the binding orientation of both ligands and showed that BVM causes pore tightening associated with structural rearrangements of residues in the SP1 helices and quenches the dynamics of the simultaneously bound IP6, consistent with stabilization of the 6-helix bundle^14–16,18,25,31^. Additionally, we uncovered previously unknown effects of BVM on arresting IP6 dynamics and attenuating side chain motions of the CA_CTD_-SP1 residues that interact with IP6, which collectively might contribute to preventing protease access to the CA-SP1 cleavage site. Furthermore, we discovered that BVM resistance in SP1-A1V and SP1-V7A variants is associated with loss of MI binding or loss of a stable 6-helix bundle conformation, respectively.

In addition to providing important molecular insights into BVM-mediated effects for Gag maturation, our study also represents a major technological advance in MAS NMR, beyond commonly used approaches for characterization of bound ligands requiring their ^13^C isotopic labeling. This was possible through judicious exploitation of ^1^H resonances of BVM as well as ^1^H and ^31^P resonances of IP6. Moreover, deuteration of CA_CTD_-SP1 and dREDOR-filtered experiments proved critical to this end. Finally,^13^C isotopic dilution was not necessary for the present MAS NMR structures of higher-order assemblies.

Taken together, the findings presented here not only elucidate the atomic details of BVM binding to HIV-1 CA_CTD_-SP1 and inform on BVM-resistance mechanisms, but also may suggest new strategies for the design of more-potent next-generation MIs.

## Methods

### Protein expression and purification

The expression plasmid for the CA_CTD_-SP1 fragment of HIV-1 strain NL4-3 Gag containing a non-cleavable His-tag and mutation P373T (SP1-P10T) was described previously^13^. The SP1-V7A and A1V mutations were introduced into the construct by Quikchange mutagenesis (Agilent). The SP1-T10 sequence polymorph exhibits the same infectivity and BVM antiviral activity (IC_50_) profiles as the SP1-P10, as shown in Supplementary Fig. 14. Proteins were expressed in transformed *E. coli* BL21 (DE3) cells, which were grown in a shaker incubator at 37 °C until mid-log phase (OD_600_ of 0.6-1) and then induced with 1 mM IPTG overnight at 18 °C. Expression of U-^13^C,^15^N enriched and U-^13^C,^15^N,^2^H enriched CA_CTD_-SP1 proteins had additional pre-culturing steps to slowly adapt the cells from rich medium to minimal medium, and performed as reported previously^32,33^. Cells were harvested by centrifugation and stored at −80 °C until use.

Protein purification was performed as previously described^13^. In brief, bacterial pellets were resuspended in 50 mM Tris, pH 8.3, 1 M LiCl, containing protease inhibitor tablets (Roche) and supplemented with 0.3% (w/v) deoxycholate. Cells were lysed by incubation with lysozyme, followed by sonication. Lysates were clarified by centrifugation, filtered, and incubated with Ni-NTA agarose resin (Qiagen) for 30 min at 4 °C. Unbound fractions were washed away, and bound protein was eluted by a step gradient of 15 mM imidazole to 300 mM imidazole. Protein was purified to homogeneity using anion exchange chromatography in 20 mM Tris, pH 8.0, 0.5 M NaCl. Pure protein was concentrated to 10 mg/mL, flash-frozen in liquid nitrogen, and stored at −80 °C until use.

### Buffer exchange

Two sets of U-^13^C,^15^N,^2^H-CA_CTD_-SP1/IP6 samples were prepared, one in Buffer A (20 mM Tris, pD 8.0, 0.5 M NaCl; made from 1 M Tris stock at 99.9% purity in D_2_O (Cambridge) and pD adjusted with deuterium chloride (Sigma) prepared in D_2_O at 99.9% purity (Cambridge)) and the second in Buffer B (20 mM Tris, pH 8.0, 0.5 M NaCl). The protein samples in Buffer A were prepared by buffer exchange as follows: 0.5 mL protein at 10 mg/mL was diluted into 10 mL with Buffer A then re-concentrated via centrifugation, 4 times. After exchange, the samples were recovered in a final volume 0.5 mL, with concentrations between 9–10 mg/mL.

### Protein assembly and sample preparation

Proteins were assembled with 1.6 mM IP6 (Sigma-Aldrich) and 1.4 mM BVM (Sigma-Aldrich), in a final reaction volume of 1 mL. Some samples that were used to obtain or confirm resonance assignments were assembled by mixing protein with equal volume of 1.5 M ammonium sulfate (Sigma-Aldrich), 0.1 M Tris, pH 8.5. Assemblies were incubated overnight. Optimal assemblies were obtained at 16-20 °C incubation temperatures. U-^13^C,^15^N labeled assemblies (~50 mg protein) were centrifuged at 10000 x *g* and packed in 3.2 mm Bruker thin-walled rotors. Buffer exchanged protein assemblies (~17 mg protein) were centrifuged at 10000 x *g* and packed in 1.9 mm Bruker rotors.

### BVM antiviral activity and particle infectivity

The activity of BVM against SP1-P10 and the SP1-T10 derivative was determined essentially as reported previously^29^. Briefly, HEK 293 T cells (ATCC, Cat# CRL-3216) were transfected with pNL4-3/SP1-P10 or pNL4-3/SP1-T10 molecular clones, and the transfected cells were treated with 24 concentrations of BVM ranging from 0 to 10 μM. Virus-containing supernatants were harvested, normalized for reverse transcriptase (RT) activity, and used to infect the TZM-bl indicator cell line. Infectivity data were analyzed with GraphPad Prism 7 from three independent experiments. Curves were fit using nonlinear regression as log(inhibitor) versus normalized response, with a variable slope using a least-squares (ordinary) fit.

To measure the relative infectivity of SP1-P10 vs. the SP1-T10 derivative, HEK 293 T cells were transfected with the pNL4-3/SP1-P10 or pNL4-3/SP1-T10 molecular clones. Virus-containing supernatants were harvested, normalized for RT activity, and used to infect the TZM-bl indicator cell line. Luciferase activity was measured at 2 days postinfection. The specific infectivities are presented relative to those of the SP1-P10 (100%). Error bars indicate standard deviations (*n*  =  4 independent assays performed in duplicate) (Supplementary Fig. 14).

### MAS NMR spectroscopy

MAS NMR experiments on U-^13^C,^15^N-CA_CTD_-SP1/BVM/IP6, U-^13^C,^15^N-CA_CTD_-SP1/IP6, U-^13^C,^15^N-CA_CTD_-SP1/BVM/SO_4_, U-^13^C,^15^N-CA_CTD_-SP1/SO_4_ crystalline arrays were performed on 20.0 T Bruker AVIII spectrometer outfitted with a 3.2 mm E-Free HCN probe. The MAS frequency was 14 kHz controlled to within ±10 Hz by a Bruker MAS controller. The actual sample temperature was maintained at 4 ± 1 °C throughout the experiments and at −10 ± 1 °C for some specific experiments using the Bruker temperature controller. The Larmor frequencies were 850.4 MHz (^1^H), 213.9 MHz (^13^C), and 86.2 MHz (^15^N) at 20.0 T. The typical 90° pulse lengths were 2.6-3.0 μs for ^1^H and 4.3-4.5 μs for ^13^C, and 4.2-4.7 μs for ^15^N. The ^1^H-^13^C and ^1^H-^15^N cross-polarization employed a linear amplitude ramp of 90-110% on ^1^H, and the center of the ramp matched to Hartmann-Hahn conditions at the first spinning sideband, with contact times of 0.7-1.5 ms and 1.0-1.7 ms, respectively. Different 2D combined R2_n_^ν^-driven (CORD)^34^ mixing times such as 10 ms, 50 ms, 100 ms, were applied for different experiments and ^1^H field strength during CORD was 14 kHz. Band-selective magnetization transfer from ^15^N to ^13^C contact time was 6.0-6.5 ms. SPINAL-64^35^ decoupling (83-95 kHz) was used during the evolution and acquisition periods.

MAS NMR experiments were also performed on 14.1 T Magnex/Bruker AVIII spectrometer outfitted with a 3.2 mm E-Free HCN probe. The Larmor frequencies were 599.8 MHz (^1^H), 150.8 MHz (^13^C), and 60.7 MHz (^15^N) at 14.1 T. The typical 90° pulse lengths were 2.6-3.0 μs for ^1^H and 4.0-4.7 μs for ^13^C, and 4.2-4.6 μs for ^15^N. The ^1^H-^13^C and ^1^H-^15^N cross-polarization employed a linear amplitude ramp of 90-110% on ^1^H, and the center of the ramp matched to Hartmann-Hahn conditions at the first spinning sideband, with contact times of 1.1-2.0 ms and 1.3-1.8 ms, respectively. Different CORD mixing times such as 25 ms, 100 ms, 250 ms, 500 ms were applied for different experiments and ^1^H field strength during CORD was 14 kHz. Band-selective ^15^N-^13^C SPECIFIC-CP contact time was 4.0-6.0 ms. SPINAL-64^35^ decoupling (80-86 kHz) was used during the evolution and acquisition periods. 2D phase-shifted ^13^C-detected proton-assisted insensitive-nuclei cross polarization (PAIN-CP)^33^ experiment was also acquired for U-^13^C,^15^N-CA_CTD_-SP1/BVM/IP6 crystalline array. During the PAIN-CP mixing period, field strengths for ^1^H, ^15^N and ^13^C channels were all 60 kHz. The length of the PAIN-CP mixing period was 4 ms.

Low temperature MAS NMR experiments of U-^13^C,^15^N-CA_CTD_-SP1/IP6 were performed on 17.6 T Bruker AVIII spectrometer outfitted with a 3.2 mm low-T E-Free HCN probe. The MAS frequency was 15 kHz controlled to within ±10 Hz by a Bruker MAS controller. The actual sample temperature was maintained at −37 ± 1 °C and −79 ± 1 °C for corresponding experiments. The Larmor frequencies were 750.1 MHz (^1^H), 188.6 MHz (^13^C), and 76.0 MHz (^15^N) at 17.6 T. The typical 90° pulse lengths were 2.5 μs for ^1^H, 3.1 μs for ^13^C, and 3.3 μs for ^15^N. The ^1^H-^13^C and ^1^H-^15^N cross-polarization employed a linear amplitude ramp of 70-100% on ^1^H, and the center of the ramp matched to Hartmann-Hahn conditions at the first spinning sideband, with contact times of 0.6 ms and 0.9 ms, respectively. CORD mixing time of 25 ms was applied for different experiments and ^1^H field strength during CORD was 15 kHz. Band-selective magnetization transfer from ^15^N to ^13^C contact time was 5.0-6.0 ms. SWFTPPM decoupling (80 kHz) was used during the evolution and acquisition periods.

MAS NMR experiments of U-^13^C,^15^N,^2^H-CA_CTD_-SP1/BVM/IP6, U-^13^C,^15^N,^2^H-CA_CTD_-SP1/IP6 crystalline arrays were performed on 20.0 T Bruker AVIII spectrometer outfitted with a 1.9 mm HCN probe. The typical 90° pulse lengths were 3.0–3.1 μs for ^1^H, 3.9-4.0 μs for ^13^C, and 3.9-4.0 μs for ^15^N. The ^1^H-^13^C and ^1^H-^15^N cross-polarization employed a linear amplitude ramp of 90-110% on ^1^H, with the center of the ramp matched to Hartmann-Hahn conditions at the first spinning sideband; contact times were 0.3-2.6 ms and 1.4-1.8 ms, respectively. The MAS frequency was 40 kHz, controlled to within ±10 Hz by a Bruker MAS controller. The actual sample temperature was maintained at 4 ± 1 °C throughout the experiments using the Bruker temperature controller. MAS NMR double-REDOR filtered experiments employed simultaneous ^1^H^13^C/^1^H^15^N REDOR dephasing periods of 5 ms, to eliminate signals from ^1^H directly bonded to ^13^C and ^15^N. The ^1^H-^13^C cross-polarization employed a linear amplitude ramp of 90-110% on ^1^H, and the center of the ramp matched to Hartmann-Hahn conditions at the first spinning sideband, with contact times of 10 ms.

MAS NMR experiments of U-^13^C,^15^N,^2^H-CA_CTD_-SP1/BVM/IP6, U-^13^C,^15^N,^2^H-CA_CTD_-SP1/IP6_,_ U-^13^C,^15^N,^2^H-CA_CTD_-SP1-V7A/BVM/IP6, U-^13^C,^15^N,^2^H-CA_CTD_-SP1-V7A/IP6, U-^13^C,^15^N,^2^H-CA_CTD_-SP1-A1V/BVM/IP6, U-^13^C,^15^N,^2^H-CA_CTD_-SP1-A1V/IP6 crystalline arrays were performed on 20.0 T Bruker AVIII spectrometer outfitted with a 1.9 mm HX probe. The typical 90° pulse lengths were 2.3-3.1 μs for ^1^H and 3.0-3.3 μs for ^13^C. The ^1^H-^13^C cross-polarization employed a linear amplitude ramp of 90-110% on ^1^H, with the center of the ramp matched to Hartmann-Hahn conditions at the first spinning sideband; contact time was 2 ms. The MAS frequency was 40 kHz and 14 kHz, controlled to within ±10 Hz by a Bruker MAS controller. The actual sample temperature was maintained at 4 ± 1 °C and at –5 ± 1 °C for some specific experiments  using the Bruker temperature controller.

^31^P solid state MAS NMR spectra were acquired on 20.0 T Bruker AVIII spectrometer outfitted with a 1.9 mm HX probe. The typical 90° pulse lengths were 2.3 μs for ^1^H and 3.0 μs for ^31^P. The ^1^H-^31^P cross-polarization employed a linear amplitude ramp of 90-110% on ^1^H, and the center of the ramp matched to Hartmann-Hahn conditions at the first spinning sideband, with contact times of 3.5 ms and 2.5 ms for out and back CP transfers, respectively. The MAS frequency was 40 kHz, controlled to within ±10 Hz by a Bruker MAS controller. The actual sample temperature was maintained at 4 ± 1 °C throughout the experiments using the Bruker temperature controller.

### Solution NMR spectroscopy

1D ^1^H and ^13^C Solution NMR spectra of BVM/DMSO-D_6_ and IP6/D_2_O were collected on a 14.1 T (^1^H Larmor frequency of 600.1 MHz) Bruker Avance spectrometer using a triple-resonance inverse detection (TXI) probe. ^1^H NMR spectra of BVM and IP6 are shown in Supplementary Fig. 12 and 15.

### Data processing

All MAS NMR data were processed using Bruker TopSpin and NMRpipe^36^. The ^13^C and ^15^N signals were referenced with respect to the external standards adamantane and ammonium chloride, respectively. ^1^H was referenced to the water peak at 4.7 ppm. ^31^P was referenced with respect to the phosphorous resonance of 85% H_3_PO_4_. The 2D and 3D data sets were processed by applying 30°, 45°, 60° and 90° shifted sine bell apodization followed by a Lorentzian-to-Gaussian transformation in both dimensions. Forward linear prediction to twice the number of the original data points was used in the indirect dimension in some data sets, followed by zero filling. The 2D CH HETCOR and dREDOR-HETCOR data of U-^13^C,^15^N,^2^H-CA_CTD_-SP1 samples were processed with gaussian apodization and quadrature baseline correction.

### MAS NMR chemical shift and distance restraints assignment

All the spectra were analyzed using CCPN^37^ and NMRFAM-Sparky^38,39^. The superposition of U-^13^C,^15^N-CA_CTD_-SP1/BVM/IP6, U-^13^C,^15^N-CA_CTD_-SP1/IP6 and, U-^13^C,^15^N-CA_CTD_-SP1/BVM/SO_4_ 2D CORD and 2D NCACX spectra at 50 ms shows no significant chemical shift differences(Supplementary Fig. 3). Chemical shift assignments (intra-residue/sequential assignments) were performed de novo on the CA_CTD_-SP1 crystalline arrays using numerous solid-state NMR data sets of U-^13^C,^15^N-CA_CTD_-SP1/BVM/SO_4_: 2D CORD, 2D and 3D dipolar-based NCACX and NCOCX, 3D CONCA, and J-based 2D direct-INADEQUATE. The backbone ^15^N and carbonyl ^13^C chemical shifts assignments of residues in the SP1 tail were performed on the basis of the 2D NCACX and NCOCX spectra of U-^13^C,^15^N-CA_CTD_-SP1/IP6 at low temperature (−37 °C and −79 °C). The de novo assignments of inter-residue ^13^C-^13^C, ^15^N-^13^C correlations were obtained for U-^13^C,^15^N-CA_CTD_-SP1/BVM/IP6 using 2D CORD spectra (100, 250, and 500 ms mixing times) and 2D NCACX (50 ms mixing time) and PAIN-CP. The ^13^C-^1^H and ^15^N-^1^H inter-residue correlations were obtained using ^1^H-detected CH and NH HETCOR spectra, respectively. On the basis of all spectra, for 64 residues all ^13^C and ^15^N backbone and side chain resonances were assigned and for 92 residues complete backbone assignments were obtained. For another 28 residues, complete backbone and partial side chain assignments were achieved, and 6 more residues had partial backbone and partial side chain assignments. For 4 residues, P147, R173, V181, and M245 (SP1-M14), no resonance assignments are available since the corresponding peaks were either missing due to dynamic disorder or overlapped with other resonances. For 84 residues, amide proton assignments (HN) were completed and, for 29 of these, backbone proton assignments (HN and Hα) were also obtained. Additionally, multiple side chain proton chemical shifts of various residues were assigned unambiguously.

BVM and IP6 proton correlations to protein resonances were assigned in 2D HC CP HETCOR, dREDOR-HETCOR, and 2D (H)PH HETCOR spectra of U-^13^C,^15^N,^2^H-CA_CTD_-SP1/BVM/IP6 and U-^13^C,^15^N,^2^H-CA_CTD_-SP1/IP6. All the samples and their experimental conditions are summarized in Supplementary Table 1. The number of cross peaks assigned in various spectra for each sample are summarized in Table 1. Chemical shifts for CA_CTD_-SP1 (crystalline array) are summarized in Supplementary Data 1.

### Determination of force field parameters for BVM and IP6

The initial coordinates of the IP6 molecule were extracted from the X-ray structure of HIV-1 immature CTD-SP1 hexamer in complex with IP6 (PDB: 6BHR)^22^, and the initial coordinates of BVM were obtained from ChemSpider (ID: 403003;^40,41^ both carboxyl groups were deprotonated.

The initial force field parameters of IP6 were derived by analogy following the CGENFF protocol^42^. The penalties for derived IP6 parameters and charges were all less than ten, indicating good analogy with the available atom types present in CGENFF^42^. Thus, the IP6 CGENFF parameters were directly used in structure calculations. BVM CHARMM force field parameters (Supplementary Fig. 16) were derived utilizing CGenFF. The partial charges and bonded interactions of BVM that were assigned a penalty score greater than ten were refined at the QM level (Supplementary Fig. 16a). The fitting evaluation between the QM potential energy surface (PES) and the CGenFF-derived Molecular Mechanics (MM) surfaces also resulted in a considerably large Root Mean Squared Error (RMSE) (Supplementary Fig. 16c). This implies that it is required to modify problematic parameters. The Force Field ToolkitM2.1^43^ (FFTK) in VMD1.9.4^44^ and Gaussian16^45^ at the MP2/6-31 G* and B3LYP/6-31 G* level of theory were used for parameter optimization. To modify the Molecular Mechanics Force Field^46^ (MMFF) parameters for the entire molecule, we used the molecule fragmentation approach^42^. First, BVM was divided into three fragments (fragments 1–3) to separate the high penalty regions (Supplementary Fig. 16b). However, one of the fragments (fragment 2) was parameterized by CGenFF with no penalty score, as shown in Supplementary Fig. 16b, and, therefore for fragment 2, we accepted CGenFF parameter without any modification. Carbon atoms at the cut points were capped with methyl groups for fragments 1 and 3. The molecular structure of these two fragments was optimized using Gaussian 16^45^. After geometry optimization at B3LYP/6-31 G* level of theory, parameters refinement proceeded in three steps.

1. First, the partial charges of atoms in BVM were optimized based on QM data at MP2/6-31 G* level of theory to reproduce the hydrogen bond interactions with a water molecule in various orientations. A complex is built for all hydrogen bond donors and acceptors containing a hydrogen bond interaction between a water molecule and fragments’ atoms.
2. Second, Gaussian calculations of the hessian were used to optimize the parameters of the bond length and bond angles with high penalty scores in each fragment using the scaled MP2/6-31 G* vibrational spectrum.
3. At the final step, dihedral angle scans were performed to generate potential energy surface PES at B3LYP/6-31 G* level of theory.

Upon completing all Gaussian calculations, the resulting QM data were used for modifying the MMFF parameters utilizing FFTK^43^ in VMD^44^. Moreover, all parameters were optimized toward the QM target data using the Downhill Simplex algorithm^47^. Subsequently, multiple iterations using QM data, obtained from steps 1 to 3, were performed to modify partial charges, bond lengths, bond angles, and dihedral angles. At each iteration, we updated the MMFF until the Molecular Mechanics (MM) potential energy surface of dihedral angles fitted to QM PES with relatively low RMSE. Subsequently, the dihedral angle parameters in each fragment yielded agreeable fits against the QM potentials, especially in potential energy regions with lower than ~10 kcal/mol, as shown in Supplementary Fig. 16d–e, with RSME = 0.985 kcal/mol for fragment 1 and RMSE = 1.065 kcal/mol for fragment 3. The PESs derived from QM conformational scans notably highlight the accuracy of the entire set of parameters, including charges, bond length, bond angles, and dihedral angles^42^ (Supplementary Fig. 16d–e). Finally, the three fragments were merged by removing the methyl groups to have one single BVM compound. The modified parameters with the method explained above are shown in Supplementary Fig. 17 and Supplementary Tables 2–3.

### Structure calculation of CA_CTD_-SP1 crystalline arrays in complex with BVM and IP6

#### Distance restraints

The distance restraints were obtained from assigned cross-peaks in MAS NMR spectra. Both unambiguous and ambiguous restraints were considered; however, restraints exceeding 5-fold ambiguity were not considered. Protein-protein restraints were ^13^C-^13^C, ^15^N-^13^C, ^15^N-^1^H, and ^13^C-^1^H restraints. IP6 and BVM restraints were ^1^H(IP6, BVM)-^13^C(protein) restraints and ^31^P(IP6)-^1^H(protein) restraints.

For CA_CTD_-SP1-V7A/BVM/IP6 structure calculation, the SP1-V7 position was computationally mutated to an alanine residue. The WT inter-residue ^13^C-^13^C, ^15^N-^13^C, and ^15^N-^1^H distance restraints were used, along with intra-residue ^13^C-^13^C, ^13^C-^1^H, ^31^P(IP6)-^1^H(protein) distance restraints, which were obtained from 2D CORD, 2D HC HETCOR, and 2D (H)PH HETCOR spectra of U-^13^C,^15^N,^2^H-CA_CTD_-SP1-V7A/BVM/IP6.

For all calculations, the bounds of the distance restraints are summarized in Table 2; these were set to 1.5-6.5 Å (4.0  ±  2.5 Å) and 2.0-7.2 Å (4.6  ±  2.6 Å) for intra- and inter-residue restraints, respectively, consistent with our previous studies.^48^ The Φ and Ψ dihedral restraints were predicted from TALOS-N^49^ using the experimental ^13^C and ^15^N chemical shifts from MAS NMR spectra.

#### Single-chain energy minimization

The single-chain structure of CA_CTD_-SP1 was calculated using MAS NMR distance and dihedral restraints using Xplor-NIH version 2.53^50–52^. Folding calculations were seeded from primary sequence extended strands. One thousand structures were calculated using molecular dynamics simulated annealing in torsion angle space with two successive annealing schedules and a final gradient minimization in Cartesian space. The structure calculation began with a 3500 K constant-temperature molecular dynamics run for the shorter of 800 ps or 8000 steps with the time step size allowed to float to maintain constant energy, within a tolerance. The initial velocities were randomized about a Maxwell distribution using a starting temperature of 3500 K. Following this initial molecular dynamics calculation, a simulated annealing calculation was performed where the temperature was reduced to 100 K in steps of 25 K. At each temperature, dynamics was run for the shorter of 0.4 ps or 200 steps. Force constants for distance restraints were ramped from 10 to 50 kcal mol^−1^ Å^−2^. The dihedral angle restraints were disabled for high-temperature dynamics at 3500 K but enabled during simulated annealing with a force constant of 200 kcal mol^−1^ rad^−2^. The gyration volume force constant^53^ was geometrically scaled from 0.002 to 1. The torsion angle database^54^ and HBPot^55^ were also used. After simulated annealing, the structures were minimized using a Powell energy minimization scheme.

Subsequently, the 10 lowest-energy structures were selected for further refinement where 1000 structures were refined in total. Annealing was performed at 3000 K for 10 ps or 5000 steps, whichever was completed first. The starting time step was 1 fs and was self-adjusted in subsequent steps to ensure conservation of energy. The initial velocities were randomized about a Maxwell distribution using the starting temperature of 3000 K. The temperature was subsequently reduced to 25 K in steps of 12.5 K. At each temperature, the initial default time step was 1 fs, and a 0.2 ps dynamics run was performed. Force constants for distance restraints were ramped from 2 to 30 kcal mol^−1^ Å^−2^. The dihedral restraint force constants were set to 10 kcal mol^−1^ rad^−2^ for high-temperature dynamics at 3000 K and 200 kcal mol^−1^ rad^−2^ during cooling. The gyration volume force constant^53^ was scaled from 0.002 to 1. The torsion angle database^54^ and HBPot^55^ were also used. The annealed structures were minimized using a Powell energy minimization scheme.

#### Docking

The lowest energy single-chain structure calculated as described above was subjected to rigid-body docking into the envelope of the hexamer-of-hexamers. The docking was performed using an in-house UCSF Chimera^56^ script (see Supplementary Note 1). Specifically, 42 best positions (from 7 hexamer units) for docking of single-chain structures, were identified in the map, on the basis of lowest cross-correlation values and brief visual inspection. Prior to docking, the density was prepared using the “molmap” routine in UCSF Chimera.

#### Refinement of the 7 hexamer units with BVM and/or IP6

After docking, a calculation was performed to identify the precise location of the IP6 and BVM ligands as well as to incorporate additional distance restraints between chains and hexamer units. The calculation was seeded from single-chain CA_CTD_-SP1 coordinates calculated from the experimental MAS NMR restraints (see above), together with the coordinates of BVM and/or IP6 generated as described above. The placement of the molecules inside a single hexamer was estimated by visual inspection to allow the protein-ligand distance restraints to be applied properly. The coordinates were expanded from a single hexamer to a hexamer-of-hexamers unit containing 7 hexamers (42 chains) using the symexp command in PyMol^57^.

100 structures underwent torsion angle dynamics with an annealing schedule and a final gradient minimization in Cartesian space. The force-field parameterization of the IP6 and BVM molecules were incorporated into the run via topology and parameter files, prepared specifically for Xplor-NIH. The BVM and IP6 molecules were free to move as rigid bodies during dynamics and final minimization. Two identical runs of simulated annealing starting at 3000 K were performed for 10 ps, with a time step of 1 fs. The initial velocities were randomized to achieve a Maxwell distribution at a starting temperature of 3000 K. The temperature was subsequently reduced to 25 K in steps of 25 K. At each temperature step, dynamics was run for 400 fs with an initial time step of 1 fs.

Standard terms for bond lengths, bond angles, and improper angles were used to enforce proper covalent geometry. Standard potentials were used to incorporate distance and dihedral restraints.

A cross-correlation probability distribution potential often utilized for experimental cryo-EM density^58^ enforced/conceded the overall shape and boundary of the hexamer of hexamers with the 8 Å density map used earlier for docking. The potential was restricted to backbone atoms (N, C, CA, and O) to ensure the density boundary would not influence side chain conformations.

A statistical torsion-angle potential^54^ was employed, and the gyration volume term was not included to avoid conflicts with the cross-correlation density potential. A hydrogen-bond database term, HBPot, was used to improve hydrogen-bond geometries^55^. Approximate non-crystallographic symmetry was imposed using Xplor-NIH’s PosDiffPot term, allowing the subunits of the hexamer to differ by up to 1 Å.

Force constants for distance restraints were ramped from 2 to 30 kcal/mol•Å^2^. The dihedral restraint force constants were set to 10 kcal/mol•rad^2^ for high temperature dynamics at 3000 K and 200 kcal/mol•rad^2^ during cooling. The force constants of the cross-correlation probability distribution potential were set to 50 kcal/mol during high temperature dynamics and cooling.

After the high-temperature dynamics and cooling in dihedral space, the annealed structures were minimized using a Powell energy minimization scheme in Cartesian space. The final MAS NMR bundle comprised the 5 lowest-energy structures of the 100 calculated ones.

RMSD values were calculated using routines in the Xplor-NIH (version 2.51)^50–52^. The visualizations of structural elements were batch rendered in PyMOL using in-house shell/bash scripts. Secondary structure elements were classified according to TALOS-N.

### Reporting summary

Further information on research design is available in the Nature Portfolio Reporting Summary linked to this article.

## Supplementary information

Supplementary Information

Description of Additional Supplementary File

Supplementary Data 1

Reporting Summary

## Data Availability

The MAS NMR atomic structure coordinates have been deposited in the Protein Data Bank under accession code 7R7P (single hexamer of CA_CTD_-SP1/BVM/IP6) and 7R7Q (single hexamer of CA_CTD_-SP1/IP6). MAS NMR chemical shifts have been deposited in the Biological Magnetic Resonance Data Bank under accession codes BMRB 30929 (CA_CTD_-SP1/BVM/IP6) and BMRB 30930 (CA_CTD_-SP1/IP6). The envelope of hexamer-of-hexamers of CA_CTD_-SP1 used in this study is available in the Protein Data Bank under accession code 5I4T. The initial coordinates of the IP6 structure used in this study are available in the Protein Data Bank under accession code 6BHR. Source data for viral infectivity assays are provided in this paper. Other data supporting the findings of the study, for example, scripts for structure calculations, analysis of calculation results and structure visualizations, are available from the corresponding authors upon request. Source data are provided with this paper.

## Source data

Source Data

## Code availability

The UCSF Chimera script for batch docking of MAS NMR structure are available in the supplementary information.
