# Supplementary material for: Structural basis of HIV-1 maturation inhibitor binding and activity
Source: Nat Commun. 2023 Mar 4;14:1237. doi: 10.1038/s41467-023-36569-y (PMC9985623; doi:10.1038/s41467-023-36569-y)
Supplement: Supplementary file 1 — Supplementary Information [file 41467_2023_36569_MOESM1_ESM.pdf]

Structural Basis of HIV-1 Maturation Inhibitor Binding and Activity

Sucharita Sarkar et al.

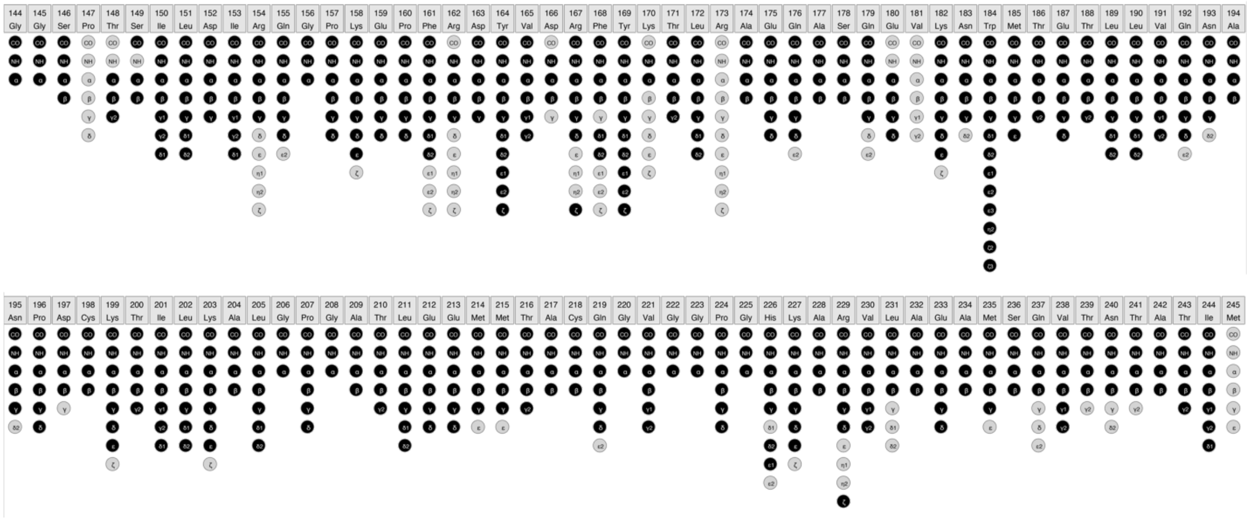

Supplementary Figure 1: Graphical summary of resonance assignments in CA$_{CTD}$-SP1 crystalline arrays. Atoms with assigned resonances are shown in black circles and those without assigned resonances are shown in gray circles.

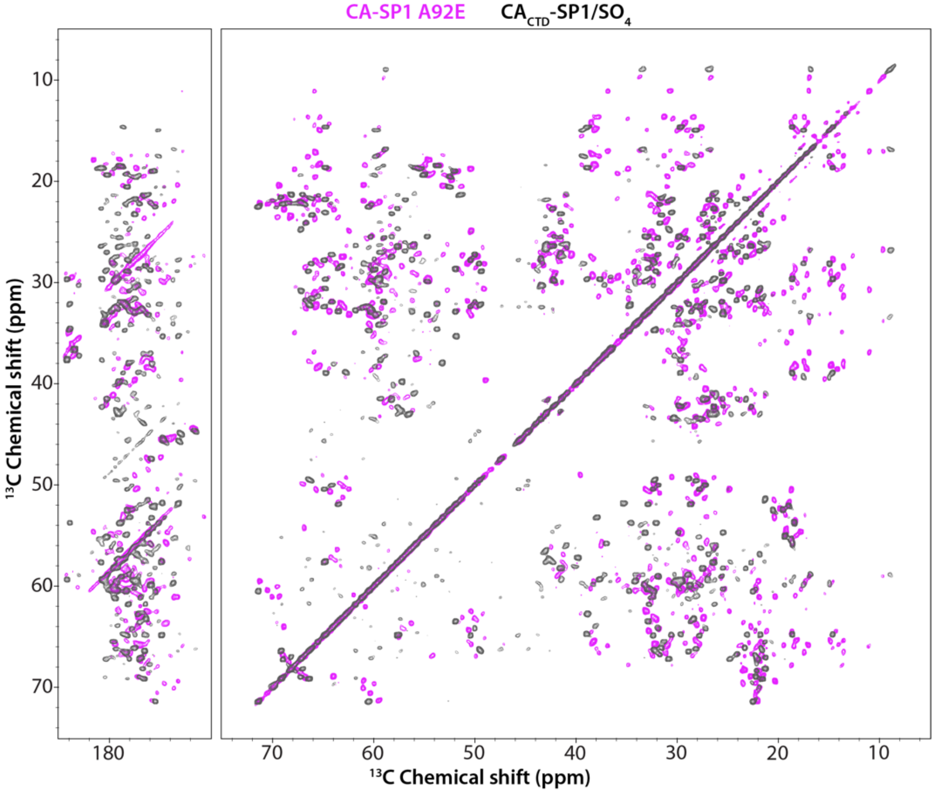

Supplementary Figure 2: Superposition of the 2D CORD spectra of CA-SP1 tubular assemblies and CA$_{CTD}$-SP1 crystalline arrays. The spectra were recorded at 20.0 T, with a MAS frequency of 14 kHz, and a CORD mixing time of 50 ms.

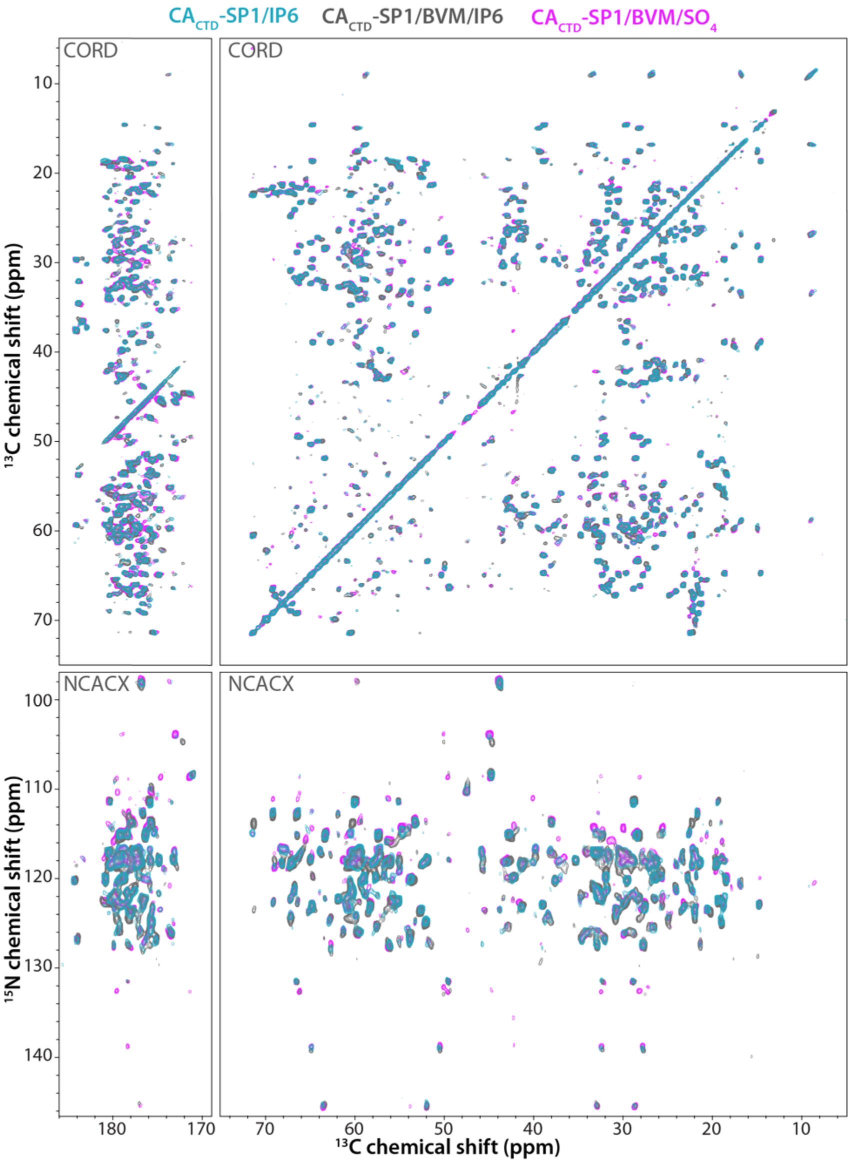

Supplementary Figure 3: Superposition of 2D CORD (top) and 2D NCACX (bottom) spectra of U-^{13}C,^{15}N-CA_{CTD}-SP1/IP6, U-^{13}C,^{15}N-CA_{CTD}-SP1/BVM/IP6, and U-^{13}C,^{15}N-CA_{CTD}-SP1/BVM/SO_4 crystalline arrays. The spectra were recorded at 20.0 T, with a MAS frequency of 14 kHz, and a CORD mixing time of 50 ms.

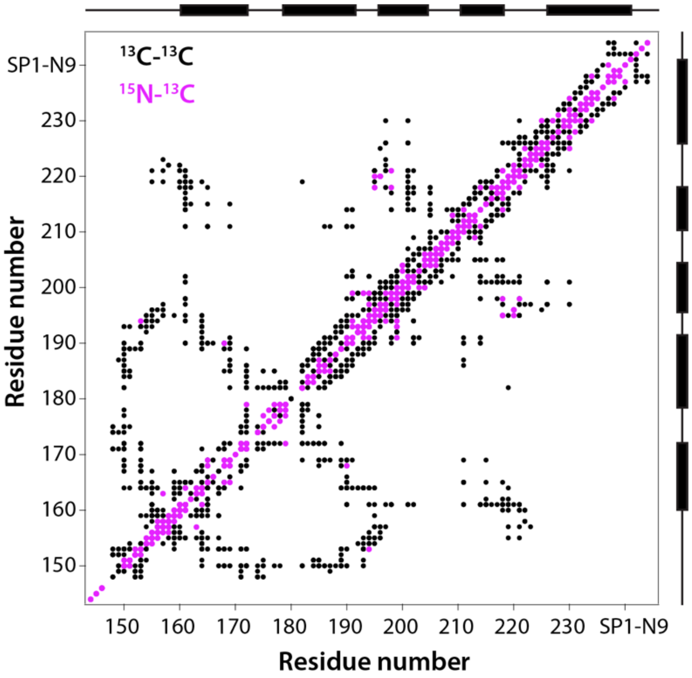

Supplementary Figure 4: ^{15}N-^{13}C and ^{13}C-^{13}C contact map of BVM and IP6 bound CA_{CTD}-SP1 crystalline arrays.

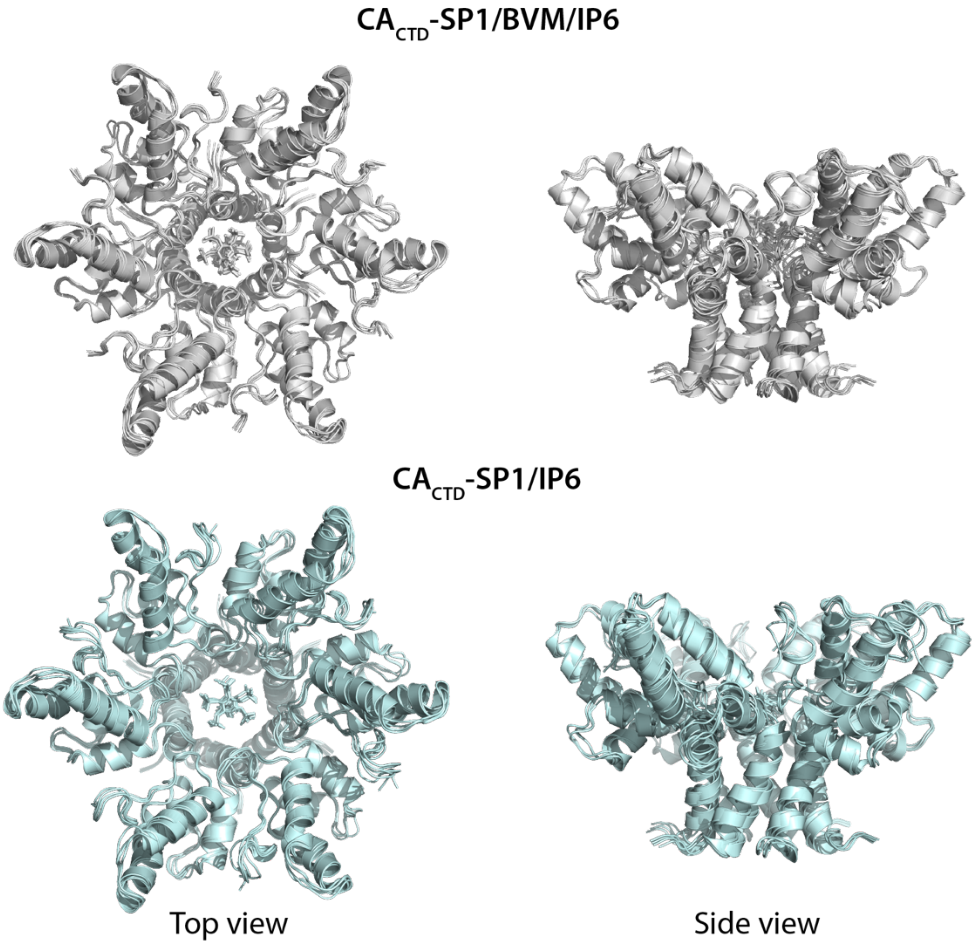

Supplementary Figure 5: Superposition of 5 lowest energy structures of central hexamer of CA_{CTD}-SP1/BVM/IP6 (top) and CA_{CTD}-SP1/IP6 (bottom) crystalline arrays.

	Restraint network	CA_{CTD}-SP1/BVM/IP6	CA_{CTD}-SP1/IP6		Restraint network	CA_{CTD}-SP1/BVM/IP6	CA_{CTD}-SP1/IP6
G144: 3 total restraints 0 long-range restraints $(\vert i-j\vert\geq5)$	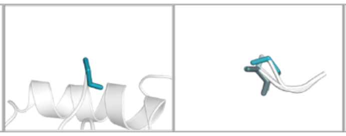			G145: 6 total restraints 1 long-range restraint $(\vert i-j\vert\geq5)$	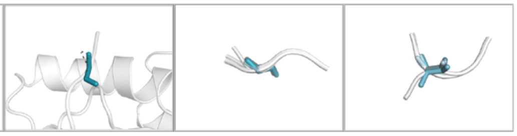		
S146: 8 total restraints 0 long-range restraints $(\vert i-j\vert\geq5)$	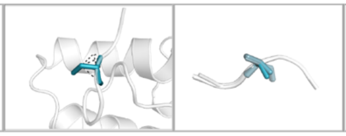			P147: 0 total restraints 0 long-range restraints $(\vert i-j\vert\geq5)$	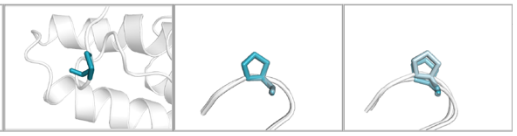		
T148: 20 total restraints 11 long-range restraints $(\vert i-j\vert\geq5)$	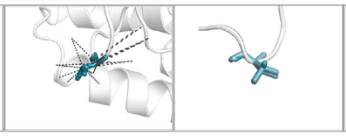			S149: 13 total restraints 2 long-range restraints $(\vert i-j\vert\geq5)$	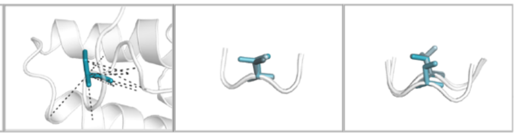		
I150: 112 total restraints 54 long-range restraints $(\vert i-j\vert\geq5)$	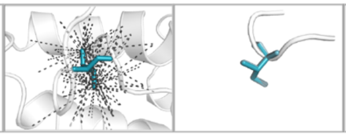			L151: 43 total restraints 11 long-range restraints $(\vert i-j\vert\geq5)$	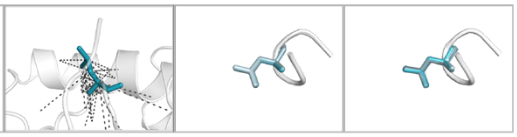		
D152: 28 total restraints 1 long-range restraint $(\vert i-j\vert\geq5)$	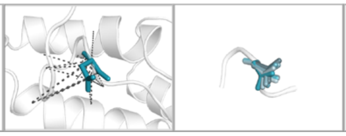			I153: 106 total restraints 55 long-range restraints $(\vert i-j\vert\geq5)$	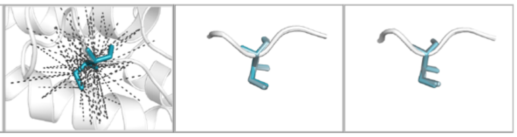		
R154: 33 total restraints 13 long-range restraints $(\vert i-j\vert\geq5)$	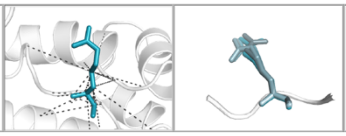			Q155: 59 total restraints 31 long-range restraints $(\vert i-j\vert\geq5)$	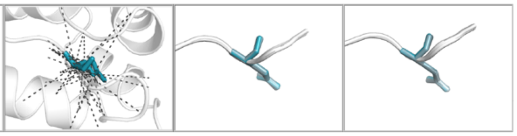		
G156: 32 total restraints 5 long-range restraints $(\vert i-j\vert\geq5)$	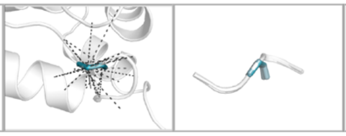			P157: 44 total restraints 7 long-range restraints $(\vert i-j\vert\geq5)$	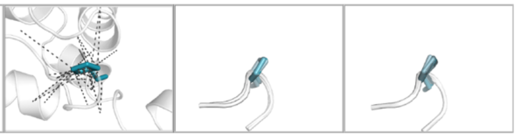		
K158: 45 total restraints 2 long-range restraints $(\vert i-j\vert\geq5)$	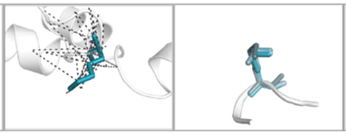			E159: 62 total restraints 9 long-range restraints $(\vert i-j\vert\geq5)$	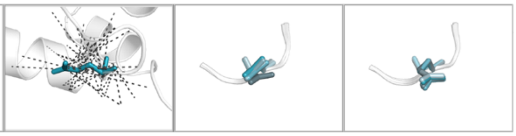		
P160: 55 total restraints 12 long-range restraints $(\vert i-j\vert\geq5)$	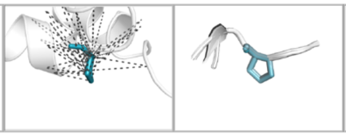			F161: 106 total restraints 63 long-range restraints $(\vert i-j\vert\geq5)$	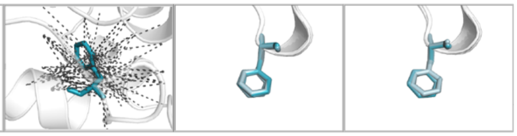		
R162: 12 total restraints 3 long-range restraints $(\vert i-j\vert\geq5)$	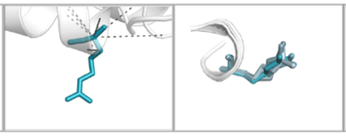			D163: 34 total restraints 1 long-range restraint $(\vert i-j\vert\geq5)$	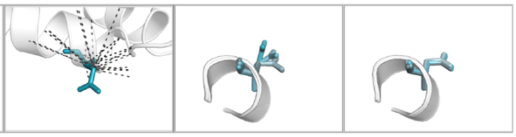		
Y164: 147 total restraints 64 long-range restraints $(\vert i-j\vert\geq5)$	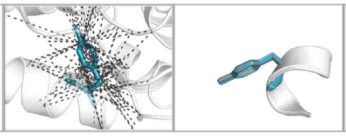			V165: 90 total restraints 18 long-range restraints $(\vert i-j\vert\geq5)$	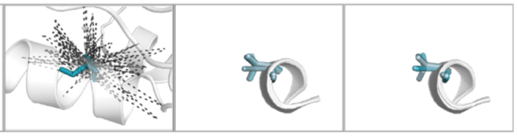		
D166: 8 total restraints 0 long-range restraints $(\vert i-j\vert\geq5)$	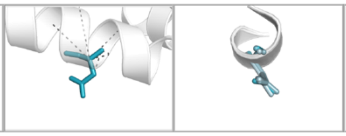			R167: 42 total restraints 18 long-range restraints $(\vert i-j\vert\geq5)$	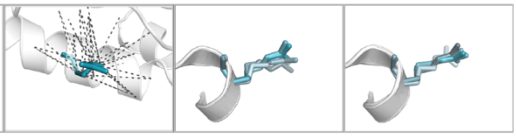		

	Restraint network	CA_{CTD}-SP1/BVM/IP6	CA_{CTD}-SP1/IP6		Restraint network	CA_{CTD}-SP1/BVM/IP6	CA_{CTD}-SP1/IP6
F168:<br>57 total restraints<br>22 long-range restraints $(\lvert i-j\rvert \geq 5)$	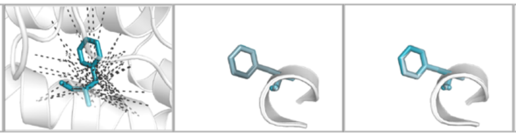			Y169:<br>58 total restraints<br>15 long-range restraints $(\lvert i-j\rvert \geq 5)$	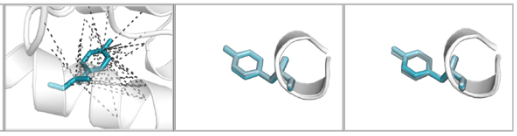		
K170:<br>1 total restraint<br>0 long-range restraints $(\lvert i-j\rvert \geq 5)$	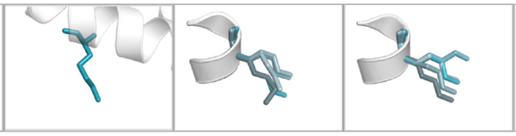			T171:<br>56 total restraints<br>13 long-range restraints $(\lvert i-j\rvert \geq 5)$	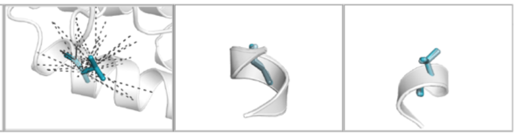		
L172:<br>68 total restraints<br>36 long-range restraints $(\lvert i-j\rvert \geq 5)$	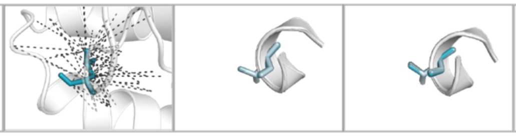			R173:<br>0 total restraints<br>0 long-range restraints $(\lvert i-j\rvert \geq 5)$	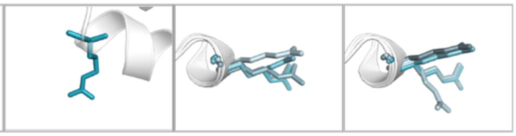		
A174:<br>23 total restraints<br>8 long-range restraints $(\lvert i-j\rvert \geq 5)$	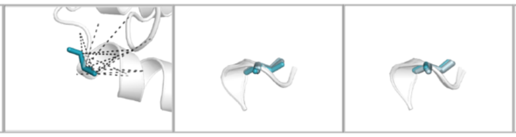			E175:<br>22 total restraints<br>8 long-range restraints $(\lvert i-j\rvert \geq 5)$	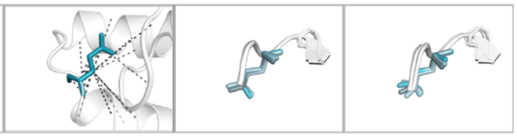		
Q176:<br>22 total restraints<br>5 long-range restraints $(\lvert i-j\rvert \geq 5)$	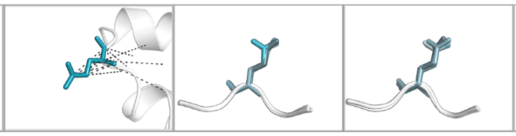			A177:<br>31 total restraints<br>14 long-range restraints $(\lvert i-j\rvert \geq 5)$	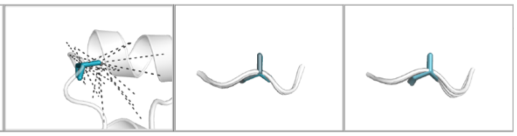		
S178:<br>25 total restraints<br>2 long-range restraints $(\lvert i-j\rvert \geq 5)$	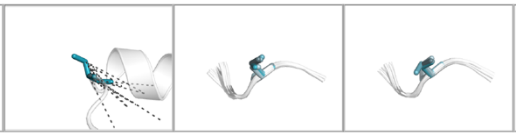			Q179:<br>25 total restraints<br>0 long-range restraints $(\lvert i-j\rvert \geq 5)$	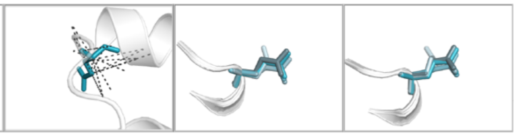		
E180:<br>6 total restraints<br>0 long-range restraints $(\lvert i-j\rvert \geq 5)$	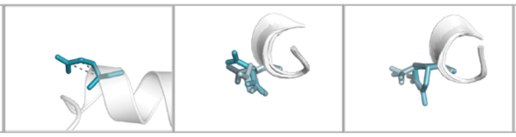			V181:<br>0 total restraints<br>0 long-range restraints $(\lvert i-j\rvert \geq 5)$	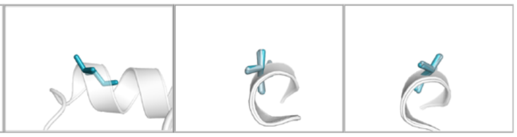		
K182:<br>69 total restraints<br>24 long-range restraints $(\lvert i-j\rvert \geq 5)$	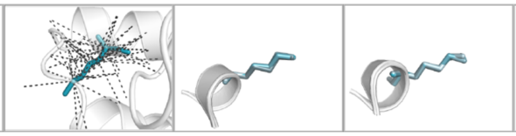			N183:<br>25 total restraints<br>2 long-range restraints $(\lvert i-j\rvert \geq 5)$	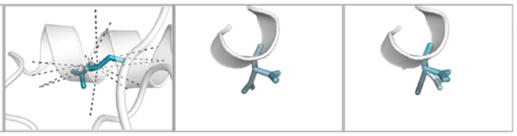		
W184:<br>61 total restraints<br>1 long-range restraint $(\lvert i-j\rvert \geq 5)$	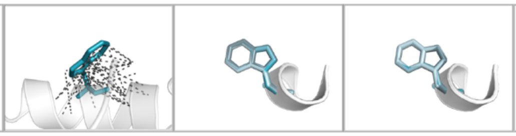			M185:<br>58 total restraints<br>24 long-range restraints $(\lvert i-j\rvert \geq 5)$	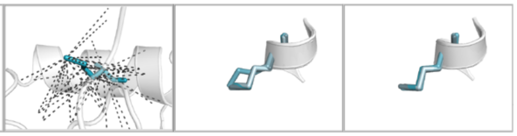		
T186:<br>41 total restraints<br>13 long-range restraints $(\lvert i-j\rvert \geq 5)$	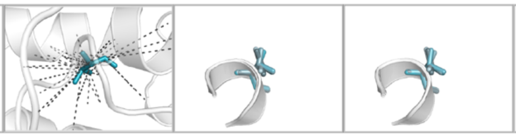			E187:<br>40 total restraints<br>2 long-range restraints $(\lvert i-j\rvert \geq 5)$	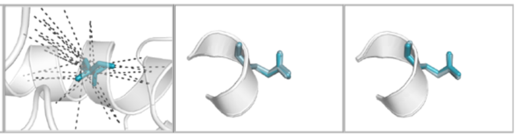		
T188:<br>34 total restraints<br>1 long-range restraint $(\lvert i-j\rvert \geq 5)$	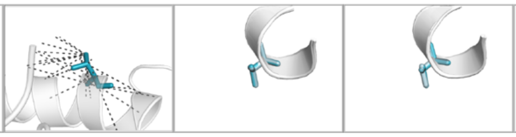			L189:<br>78 total restraints<br>35 long-range restraints $(\lvert i-j\rvert \geq 5)$	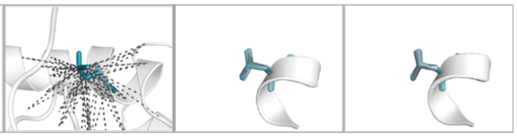		
L190:<br>96 total restraints<br>52 long-range restraints $(\lvert i-j\rvert \geq 5)$	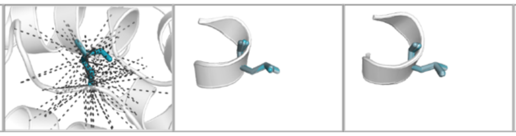			V191:<br>72 total restraints<br>33 long-range restraints $(\lvert i-j\rvert \geq 5)$	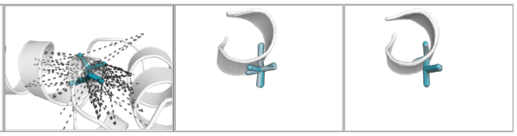		

	Restraint network	CA_{CTD}-SP1/BVM/IP6	CA_{CTD}-SP1/IP6		Restraint network	CA_{CTD}-SP1/BVM/IP6	CA_{CTD}-SP1/IP6
Q192:<br>35 total restraints<br>9 long-range restraints $(\lvert i-j\rvert \geq 5)$	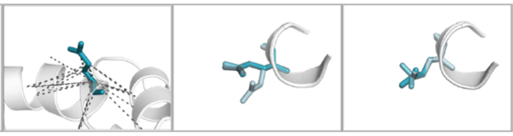			N193:<br>65 total restraints<br>26 long-range restraints $(\lvert i-j\rvert \geq 5)$	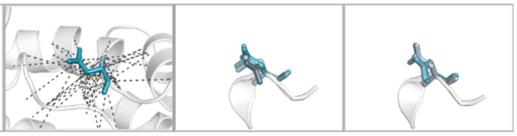		
A194:<br>73 total restraints<br>25 long-range restraints $(\lvert i-j\rvert \geq 5)$	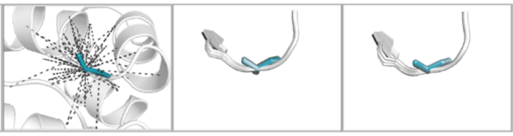			N195:<br>69 total restraints<br>19 long-range restraints $(\lvert i-j\rvert \geq 5)$	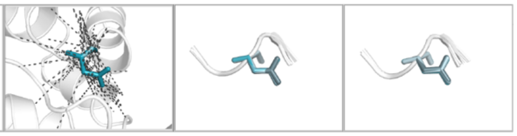		
P196:<br>54 total restraints<br>6 long-range restraints $(\lvert i-j\rvert \geq 5)$	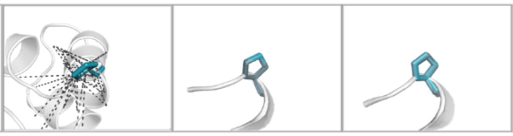			D197:<br>44 total restraints<br>12 long-range restraints $(\lvert i-j\rvert \geq 5)$	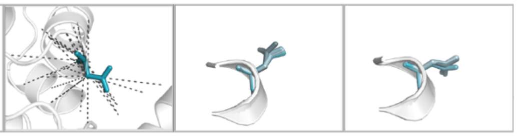		
C198:<br>71 total restraints<br>23 long-range restraints $(\lvert i-j\rvert \geq 5)$	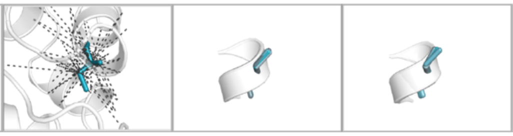			K199:<br>122 total restraints<br>34 long-range restraints $(\lvert i-j\rvert \geq 5)$	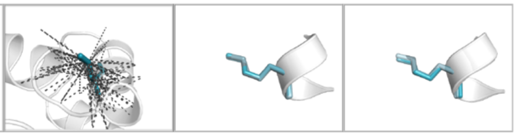		
T200:<br>78 total restraints<br>0 long-range restraints $(\lvert i-j\rvert \geq 5)$	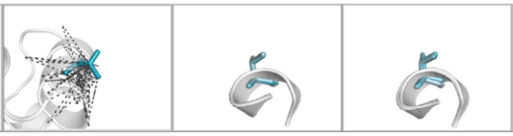			I201:<br>141 total restraints<br>46 long-range restraints $(\lvert i-j\rvert \geq 5)$	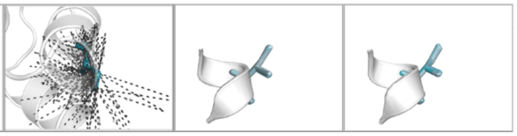		
L202:<br>68 total restraints<br>23 long-range restraints $(\lvert i-j\rvert \geq 5)$	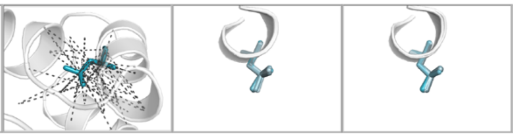			K203:<br>48 total restraints<br>2 long-range restraints $(\lvert i-j\rvert \geq 5)$	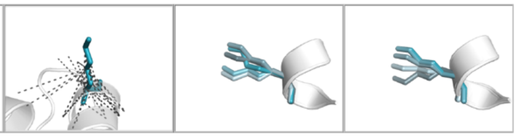		
A204:<br>39 total restraints<br>0 long-range restraints $(\lvert i-j\rvert \geq 5)$	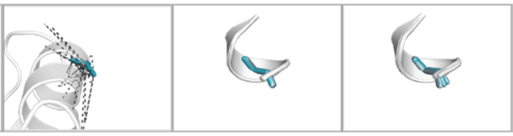			L205:<br>66 total restraints<br>23 long-range restraints $(\lvert i-j\rvert \geq 5)$	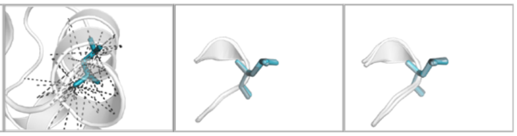		
G206:<br>30 total restraints<br>0 long-range restraints $(\lvert i-j\rvert \geq 5)$	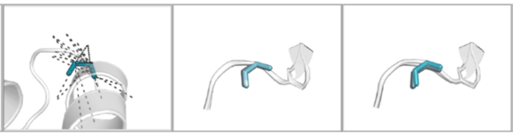			P207:<br>40 total restraints<br>0 long-range restraints $(\lvert i-j\rvert \geq 5)$	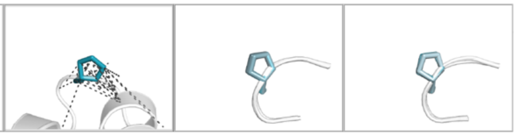		
G208:<br>12 total restraints<br>0 long-range restraints $(\lvert i-j\rvert \geq 5)$	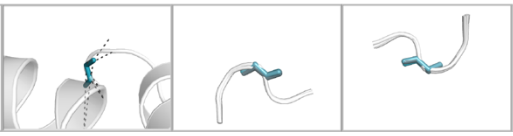			A209:<br>41 total restraints<br>3 long-range restraints $(\lvert i-j\rvert \geq 5)$	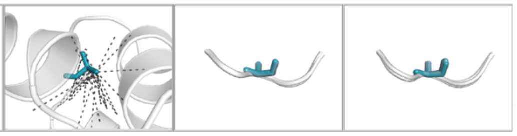		
T210:<br>62 total restraints<br>1 long-range restraint $(\lvert i-j\rvert \geq 5)$	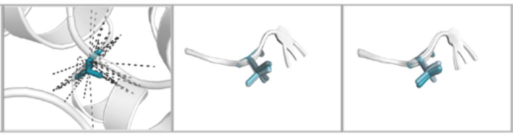			L211:<br>65 total restraints<br>16 long-range restraints $(\lvert i-j\rvert \geq 5)$	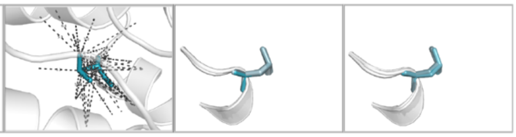		
E212:<br>34 total restraints<br>0 long-range restraints $(\lvert i-j\rvert \geq 5)$	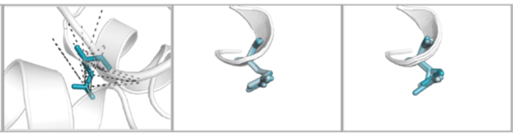			E213:<br>58 total restraints<br>9 long-range restraints $(\lvert i-j\rvert \geq 5)$	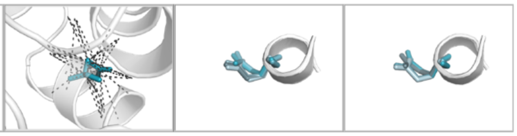		
M214:<br>64 total restraints<br>37 long-range restraints $(\lvert i-j\rvert \geq 5)$	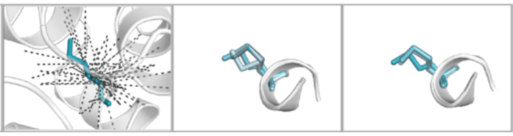			M215:<br>48 total restraints<br>10 long-range restraints $(\lvert i-j\rvert \geq 5)$	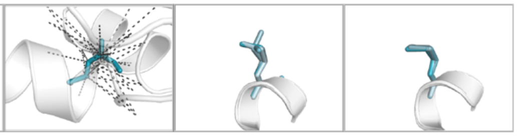		

	Restraint network	CA_{CTD}-SP1/BVM/IP6	CA_{CTD}-SP1/IP6		Restraint network	CA_{CTD}-SP1/BVM/IP6	CA_{CTD}-SP1/IP6
T216:<br>47 total restraints<br>2 long-range restraints $(\lvert i-j \rvert \geq 5)$	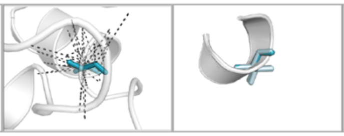			A217:<br>49 total restraints<br>11 long-range restraints $(\lvert i-j \rvert \geq 5)$	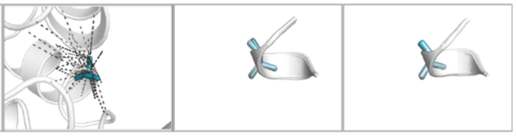		
C218:<br>73 total restraints<br>37 long-range restraints $(\lvert i-j \rvert \geq 5)$	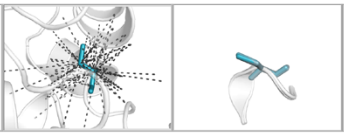			Q219:<br>51 total restraints<br>12 long-range restraints $(\lvert i-j \rvert \geq 5)$	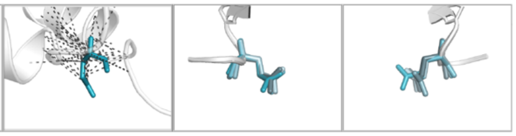		
G220:<br>33 total restraints<br>7 long-range restraints $(\lvert i-j \rvert \geq 5)$	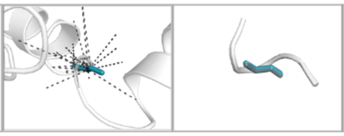			V221:<br>94 total restraints<br>53 long-range restraints $(\lvert i-j \rvert \geq 5)$	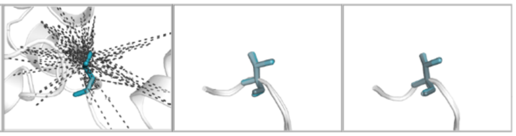		
G222:<br>23 total restraints<br>8 long-range restraints $(\lvert i-j \rvert \geq 5)$	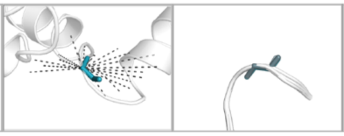			G223:<br>30 total restraints<br>0 long-range restraints $(\lvert i-j \rvert \geq 5)$	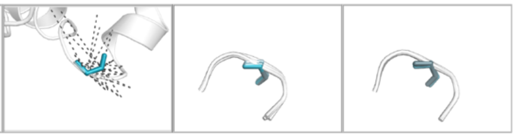		
P224:<br>55 total restraints<br>0 long-range restraints $(\lvert i-j \rvert \geq 5)$	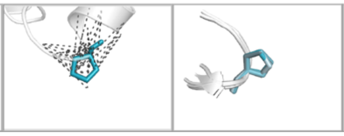			G225:<br>35 total restraints<br>1 long-range restraint $(\lvert i-j \rvert \geq 5)$	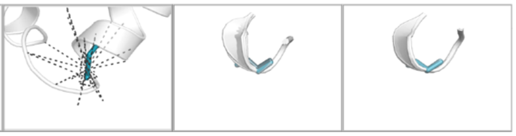		
H226:<br>109 total restraints<br>31 long-range restraints $(\lvert i-j \rvert \geq 5)$	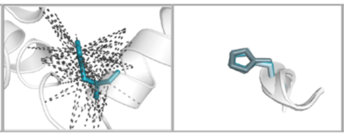			K227:<br>50 total restraints<br>7 long-range restraints $(\lvert i-j \rvert \geq 5)$	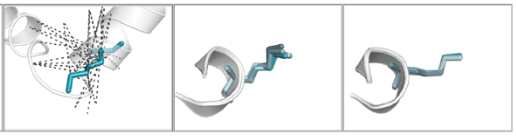		
A228:<br>28 total restraints<br>0 long-range restraints $(\lvert i-j \rvert \geq 5)$	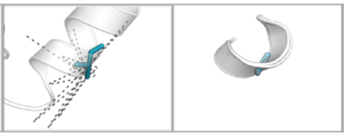			R229:<br>47 total restraints<br>0 long-range restraints $(\lvert i-j \rvert \geq 5)$	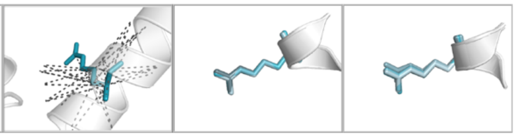		
V230:<br>63 total restraints<br>5 long-range restraints $(\lvert i-j \rvert \geq 5)$	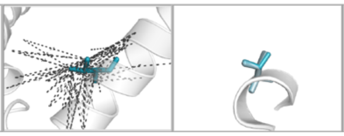			L231:<br>13 total restraints<br>0 long-range restraints $(\lvert i-j \rvert \geq 5)$	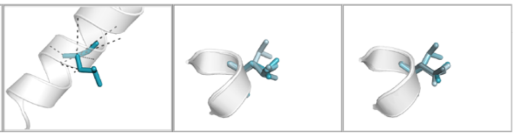		
A232: (SP1-A1)<br>31 total restraints<br>0 long-range restraints $(\lvert i-j \rvert \geq 5)$	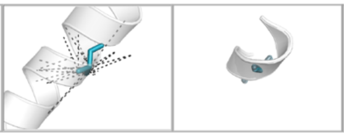			E233: (SP1-E2)<br>57 total restraints<br>0 long-range restraints $(\lvert i-j \rvert \geq 5)$	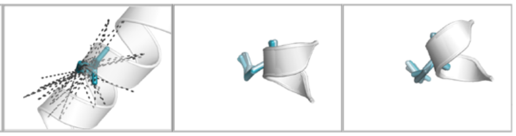		
A234: (SP1-A3)<br>36 total restraints<br>0 long-range restraints $(\lvert i-j \rvert \geq 5)$	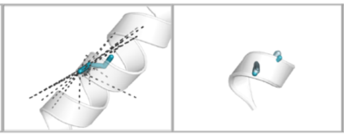			M235: (SP1-M4)<br>28 total restraints<br>1 long-range restraint $(\lvert i-j \rvert \geq 5)$	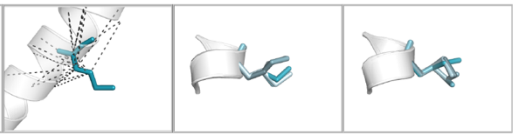		
S236: (SP1-S5)<br>11 total restraints<br>0 long-range restraints $(\lvert i-j \rvert \geq 5)$	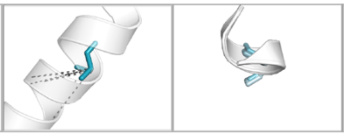			Q237: (SP1-Q6)<br>24 total restraints<br>3 long-range restraints $(\lvert i-j \rvert \geq 5)$	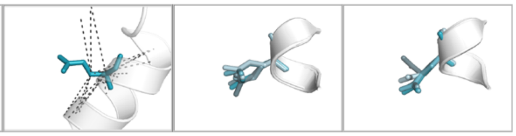		
V238: (SP1-V7)<br>38 total restraints<br>4 long-range restraints $(\lvert i-j \rvert \geq 5)$	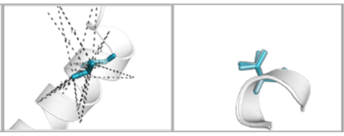			T239: (SP1-T8)<br>11 total restraints<br>0 long-range restraints $(\lvert i-j \rvert \geq 5)$	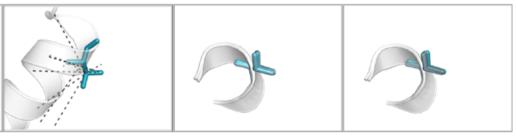		

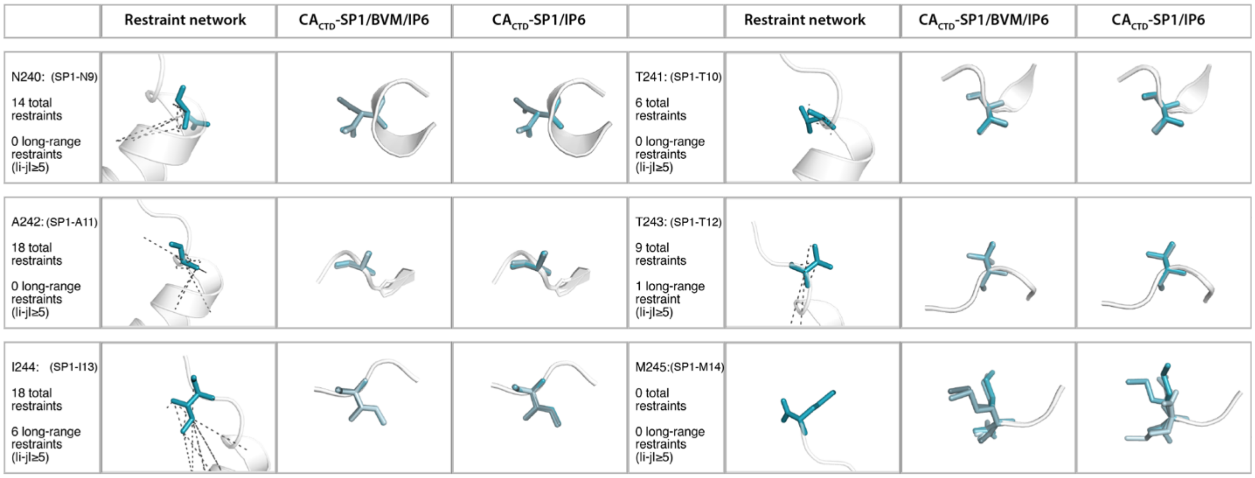

Supplementary Figure 6: Side chain conformations in the refined structure of CA$_{CTD}$-SP1/BVM/IP6 crystalline arrays. Experimental NMR intra-chain distances (gray dotted lines) are mapped onto the structure for each residue, if available.

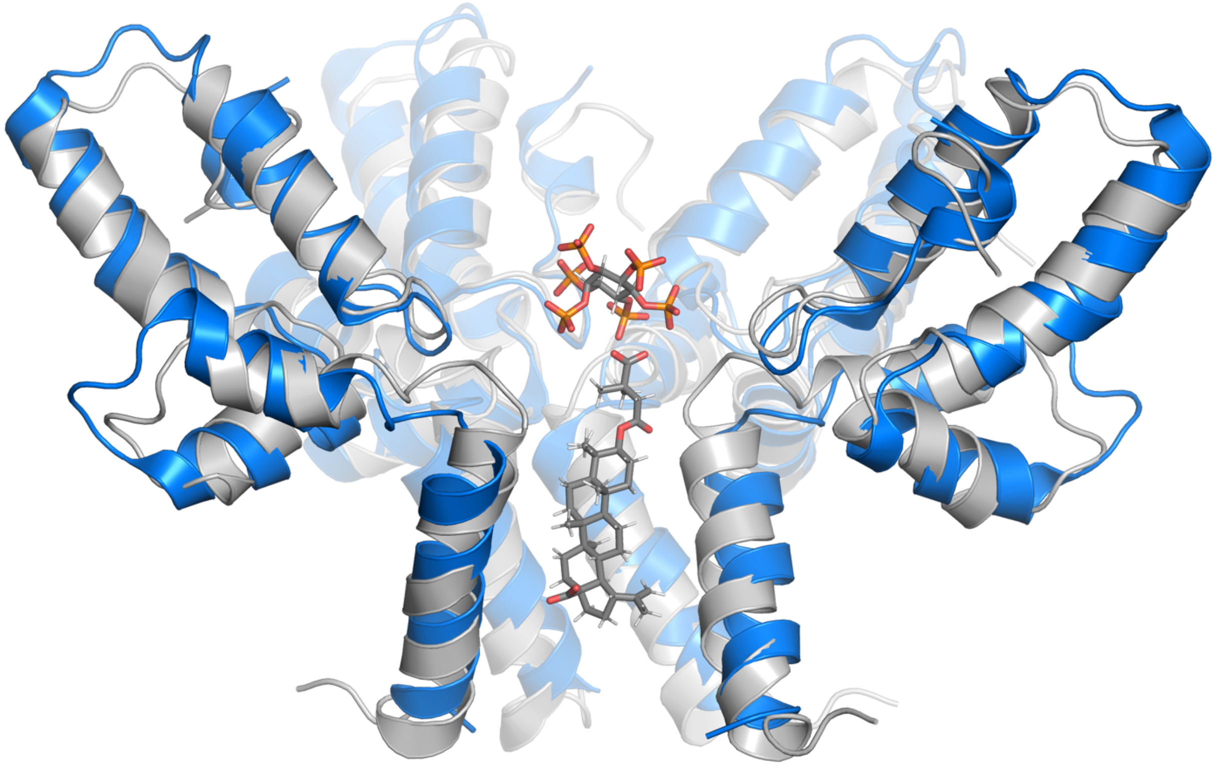

Supplementary Figure 7: Comparison of microED structure of CA_{CTD}-SP1 (PDB ID: 6N3U, blue) and MAS NMR structure of CA_{CTD}-SP1/BVM/IP6 (PDB ID: 7R7P, gray, this work).

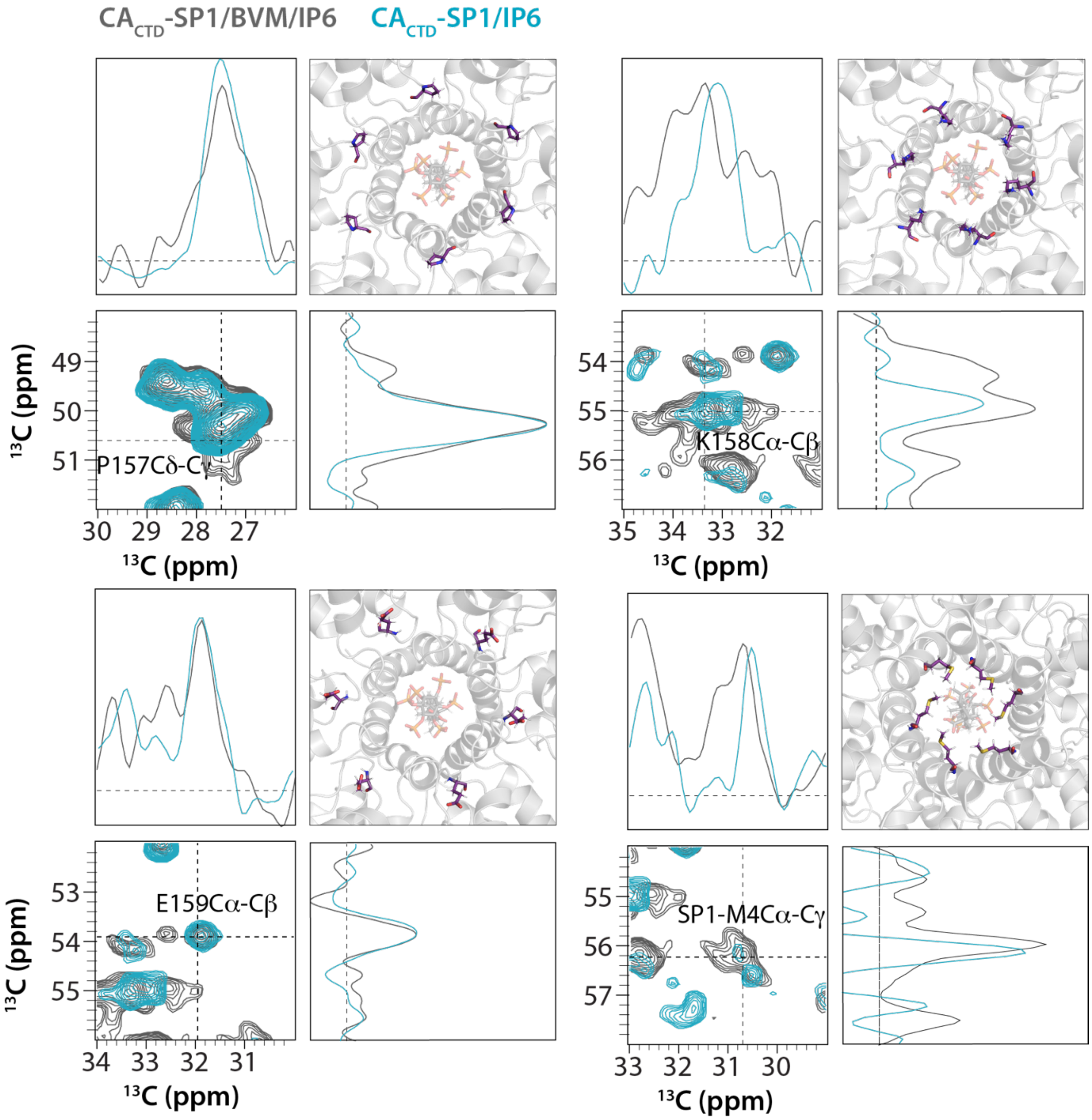

Supplementary Figure 8: BVM binding to CA$_{CTD}$-SP1 crystalline arrays induces conformational heterogeneity in the binding region. Expansions around selected regions of the CORD spectra of CA$_{CTD}$-SP1/IP6 (cyan) and CA$_{CTD}$-SP1/BVM/IP6 (gray). The spectra were recorded at 20.0 T, with a MAS frequency of 14 kHz, and a CORD mixing time of 50 ms.

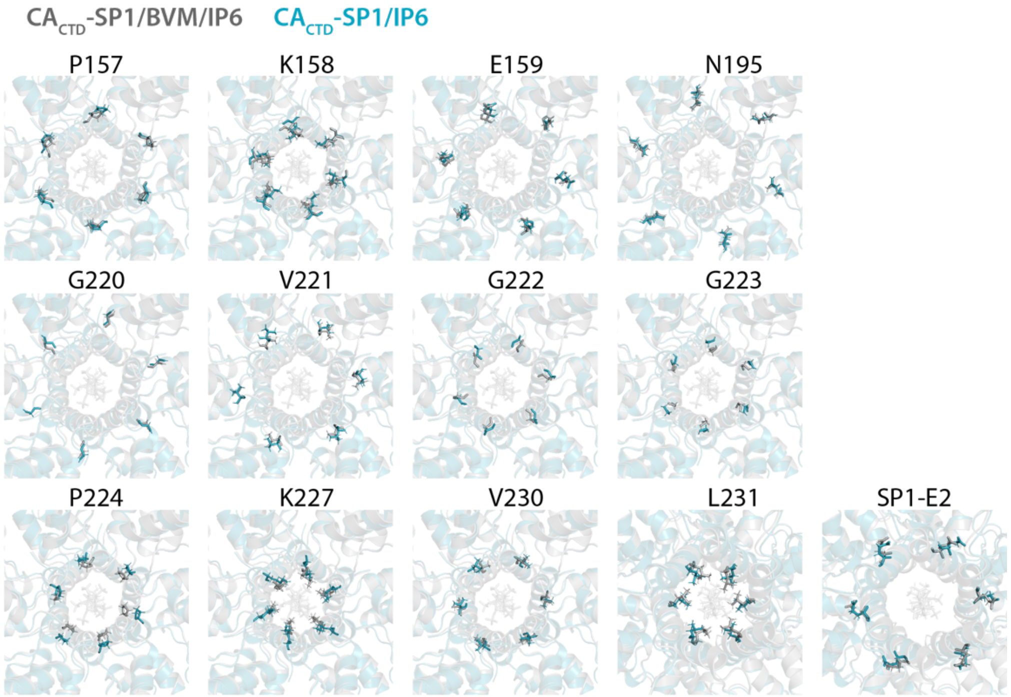

Supplementary Figure 9: Reorientation of side chains in CA$_{CTD}$-SP1 crystalline arrays induced by BVM binding. The sidechain conformations were determined based on multiple NMR distance restraints as described in Methods and discussed in the text.

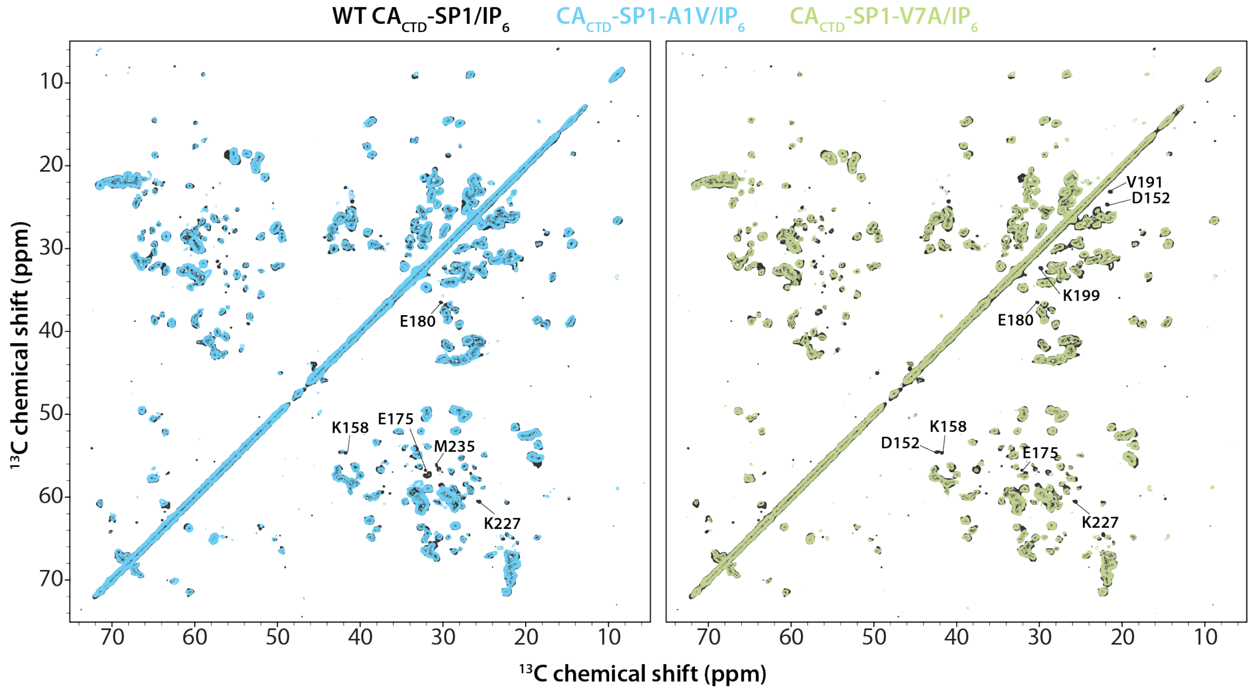

Supplementary Figure 10: Comparison of 2D CORD spectra of wild-type, A1V, V7A variant. Left: Superposition of 2D CORD spectra of U-^{13}C,^{15}N,^{2}H-CA$_{CTD}$-SP1/IP6 (black) and U-^{13}C,^{15}N,^{2}H-CA$_{CTD}$-SP1-A1V/IP6 (light blue). Right: Superposition of 2D CORD spectra of U-^{13}C,^{15}N,^{2}H-CA$_{CTD}$-SP1/IP6 (black) and U-^{13}C,^{15}N,^{2}H-CA$_{CTD}$-SP1-V7A/IP6 (light green). Selected missing peaks in the variants are labeled.

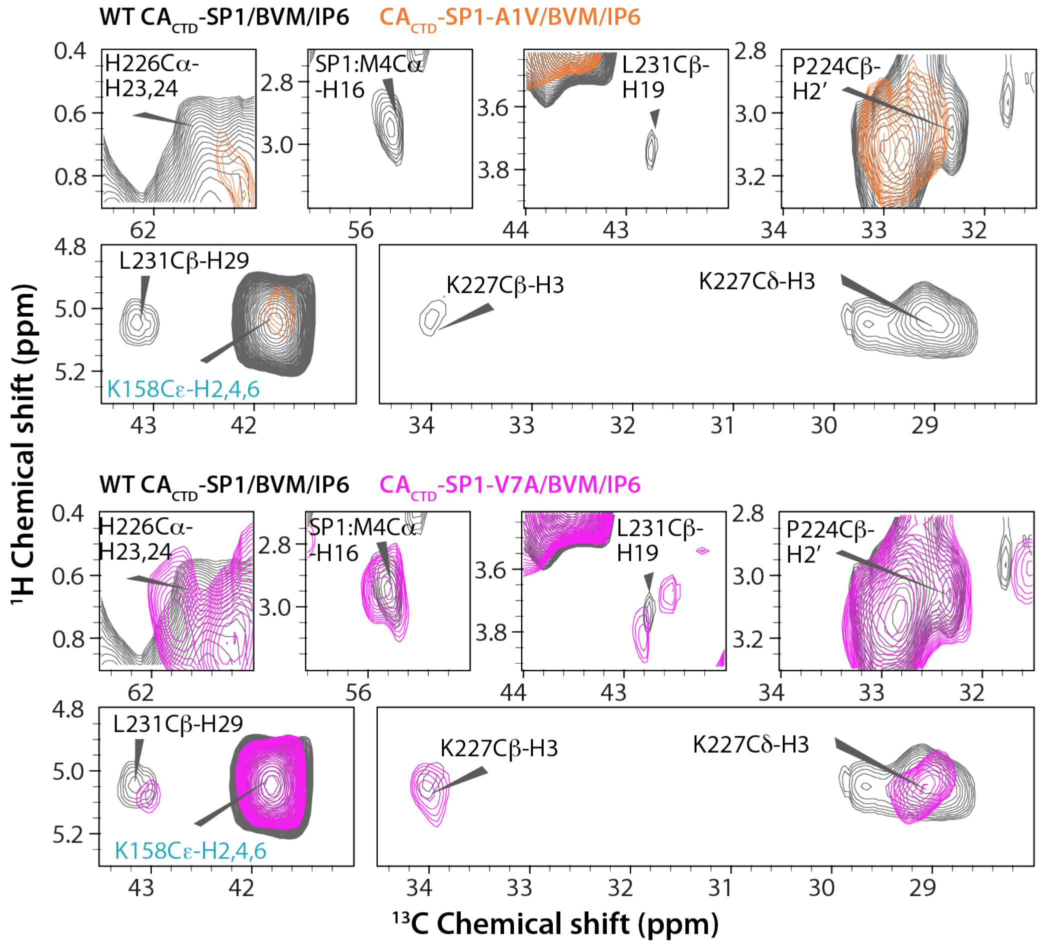

Supplementary Figure 11: BVM-protein and IP6-protein correlations in MAS NMR spectra of wild-type, A1V, V7A variant. Superposition of selected regions of 2D HC CP HETCOR spectra of U-^{13}C,^{15}N,^{2}H-CA_{CTD}-SP1/BVM/IP6 (gray) and U-^{13}C,^{15}N,^{2}H-CA_{CTD}-SP1-A1V/BVM/IP6 (orange) (top two rows), and U-^{13}C,^{15}N,^{2}H-CA_{CTD}-SP1/BVM/IP6 (gray) and U-^{13}C,^{15}N,^{2}H-CA_{CTD}-SP1-V7A/BVM/IP6 (magenta) (bottom two rows). BVM-protein (colored black) and IP6-protein (colored cyan) correlations are labelled. In A1V variant, BVM-protein correlations are missing, and IP6-protein correlation is very weak. In V7A variant, all BVM-protein and IP6-protein correlations are present.

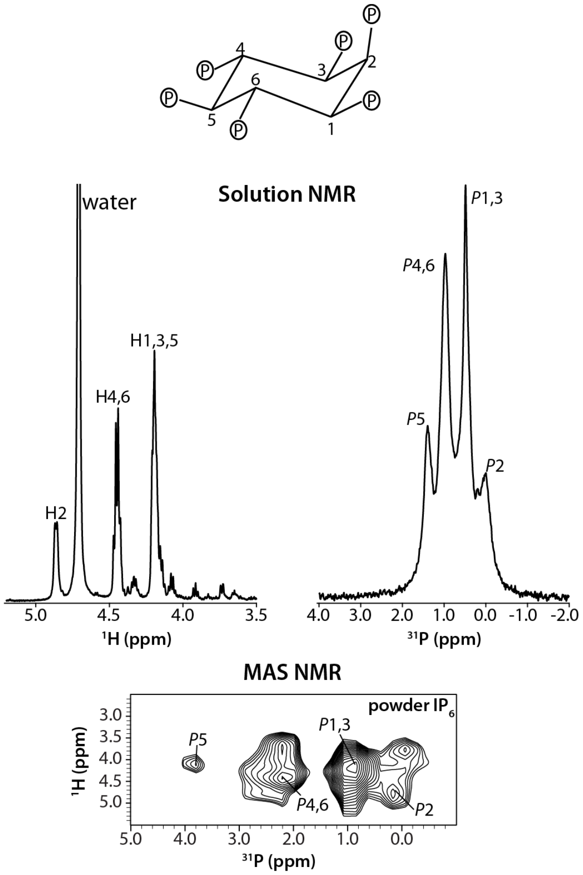

Supplementary Figure 12: 1D solution NMR and 2D MAS NMR spectra of IP6. 1D solution NMR spectra of IP6/D_2O were recorded at 14.1 T. 2D (H)PH HETCOR spectra of powder IP6 was recorded at 20.0 T.

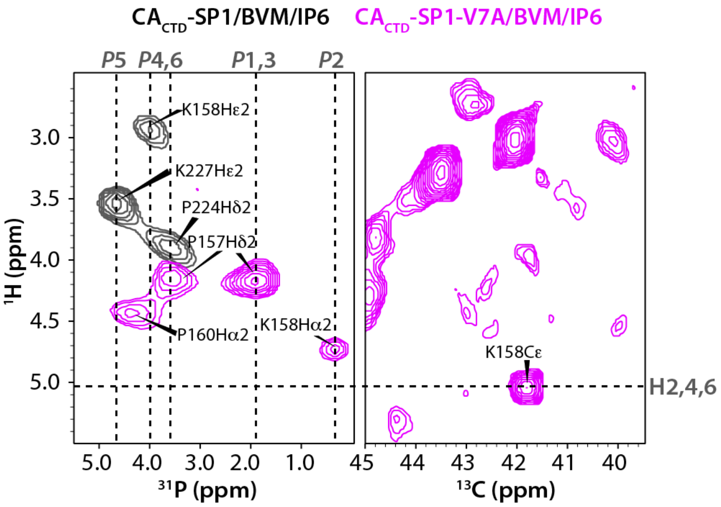

Supplementary Figure 13: MAS NMR chemical shift assignment of IP6 bound to wild-type and V7A CA_{CTD}-SP1 with BVM. a) IP6 phosphorus (*P*) and protons (H) are labeled outside the box. The left panel is overlay of 2D (H)PH HETCOR spectra of U-^{13}C,^{15}N,^{2}H-CA_{CTD}-SP1/BVM/IP6 (gray) and U-^{13}C,^{15}N,^{2}H-CA_{CTD}-SP1-V7A/BVM/IP6 (magenta). The right panel is 2D HC HETCOR spectra of U-^{13}C,^{15}N,^{2}H-CA_{CTD}-SP1-V7A/BVM/IP6 showing the H2,4,6 (IP6) – K158Cε (CA_{CTD}-SP1) correlation. This indicates that in 2D (H)PH HETCOR spectra the correlations are not from intramolecular IP6 correlations.

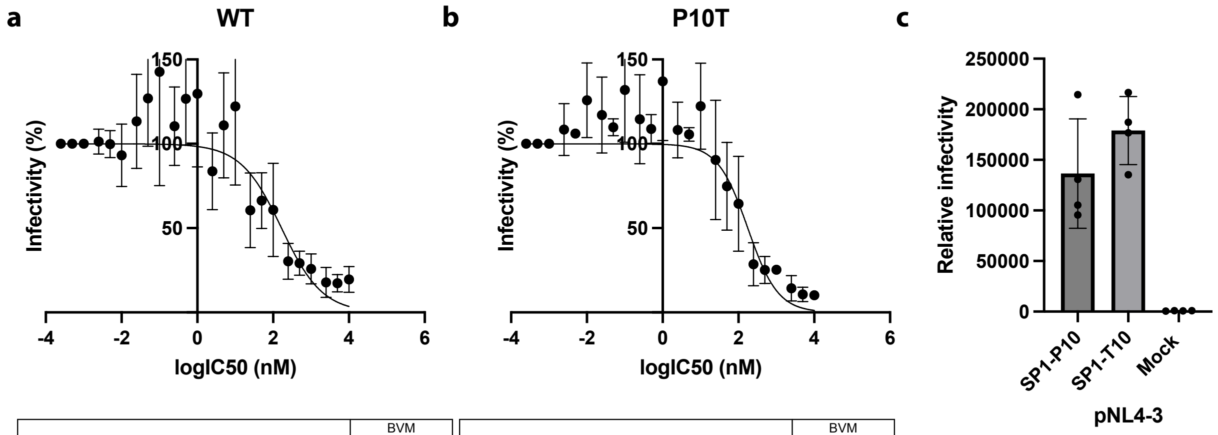

	BVM
log(inhibitor) vs. normalized response -- Variable slope	
Best-fit values	
LogIC50	2.222
HillSlope	-0.8051
IC50	166.6
95% CI (profile likelihood)	
LogIC50	1.874 to 2.606

	BVM
log(inhibitor) vs. normalized response -- Variable slope	
Best-fit values	
LogIC50	2.247
HillSlope	-1.095
IC50	176.5
95% CI (profile likelihood)	
LogIC50	2.019 to 2.495

Supplementary Figure 14: Viral infectivity and sensitivity to BVM. Antiviral activity of BVM against **a)** SP1-P10 and **b)** the SP1-T10 sequence variants. **c)** Relative infectivity of SP1-P10 and SP1-T10 sequence variants using the TZM-bl indicator cell line. N = 4 biologically independent experiments were carried out. Data are presented as mean values +/- standard deviation. Source data are provided as a Source Data file.

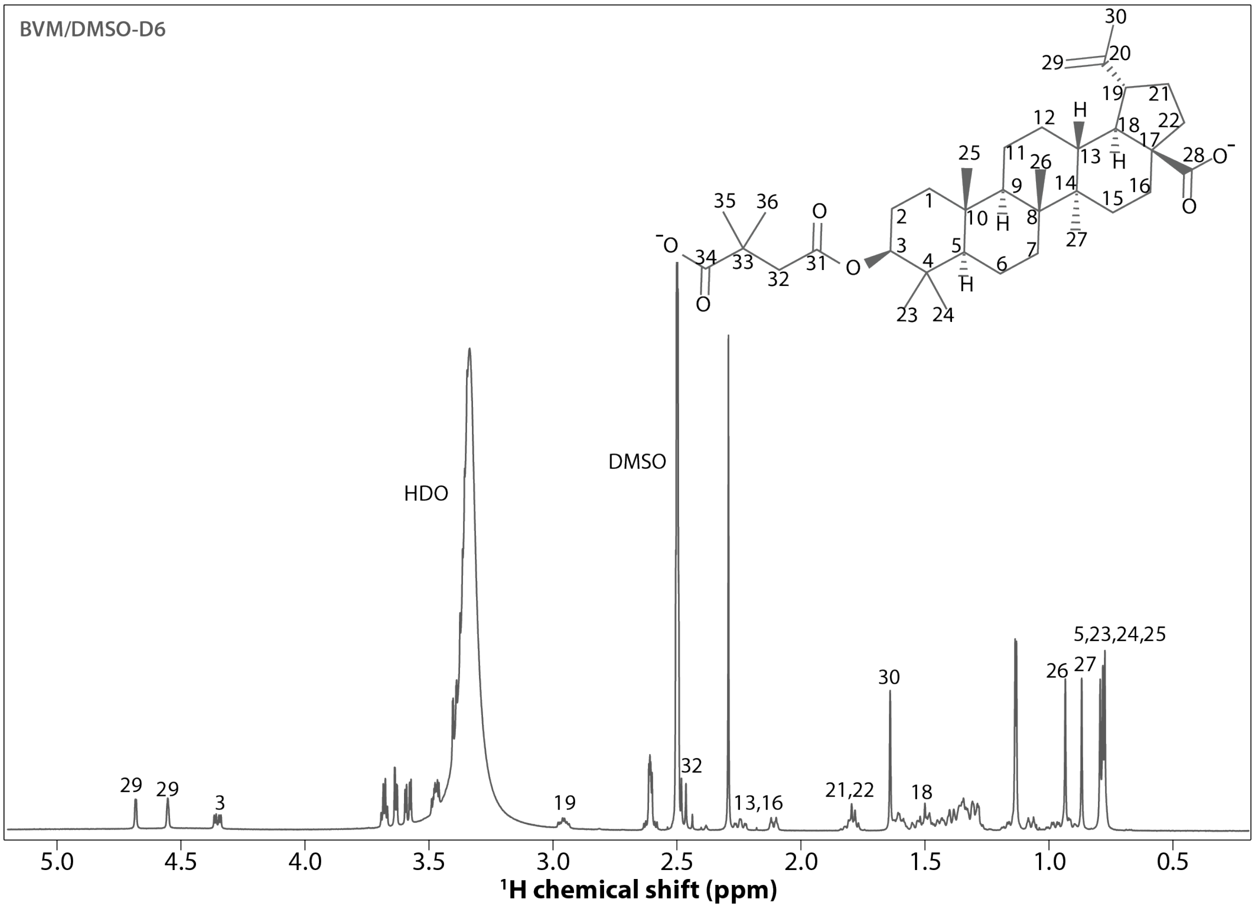

Supplementary Figure 15: ^{1}H solution NMR spectra of BVM dissolved in DMSO-D_6 (14.1 T).

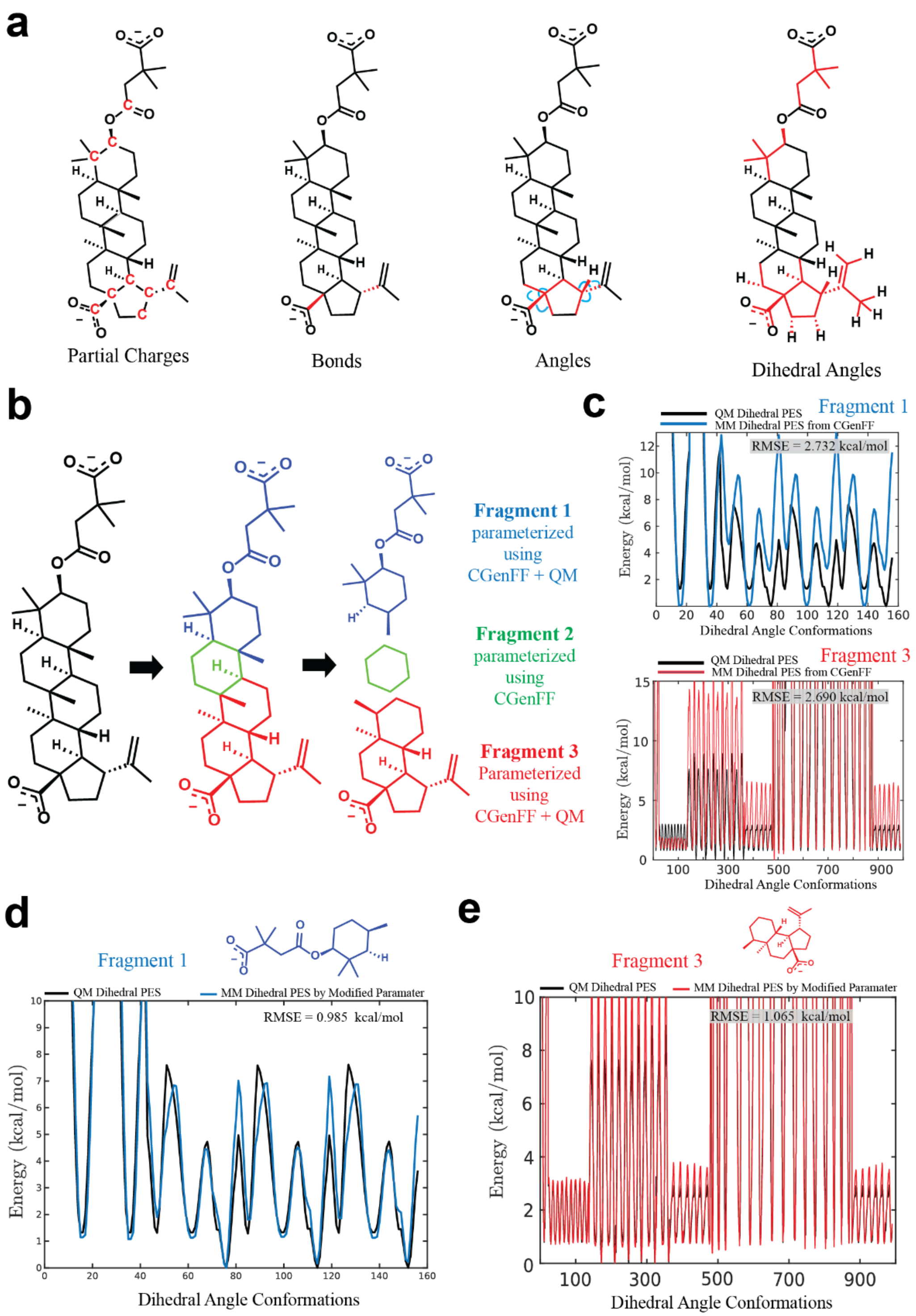

Supplementary Figure 16: BVM CHARMM force-field parameters refinement. a) The problematic parameters for BVM were identified using CGenFF. Partial charges, Bonds, Angles, and Dihedral angles with high penalty scores assigned by CGenFF and highlighted by red indicate these degrees of freedom have parameters with a penalty score greater than ten. **b)** BVM was separated into three fragments. Fragment 1 is shown in blue and consists of the dimethyl succinate moiety. Fragment 2 is shown in green, and MMFF parameters assigned by CGenFF for this fragment have a zero-penalty score. Fragment 3 is represented in red, and three out of five carbon rings of the Pentacyclic Triterpenoid moiety are included in

this fragment. Moreover, bonds were capped with a methyl group at the cut points. **c)** Fitting the QM and MM potential energy profiles derived from the CGenFF dihedral parameters for fragments 1 and 3. RMSE for the fitting of QM and CGenFF potential energy surface for fragment 1 has an RMSE = 2.732 kcal/mol, and fragment 3 has an RMSE = 2.690 kcal/mol, suggesting that these CGenFF-derived parameters do not correctly compromise with the QM level of theory. **d,e)** Fitting the QM and MM potential energy profiles of the modified dihedral parameters for fragments 1 and 3, respectively. These torsional potential energy surfaces evaluate the entire force field, namely partial charges, bonds, angles, and dihedral angles, indicating that the parameters accurately agree with the QM level of theory. After parameter refinement, the RMSE for fragment 1 and 3 resulted RMSE = 0.985 kcal/mol and RMSE = 1.065 kcal/mol, respectively.

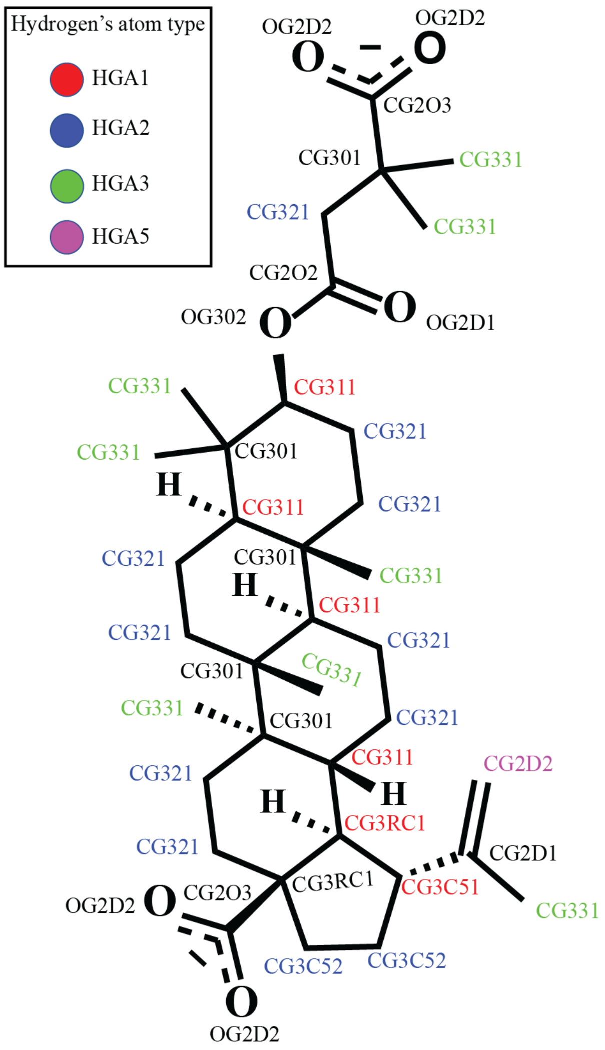

Supplementary Figure 17: BVM's atom type used for optimization. Each color represents the hydrogen atom's type which bonds to the corresponding carbon with the exact color. For example, the dihedral angle CG3C51- CG2D1-CG2D2 and purple indicate hydrogen atom HGA5 (hydrogen atom type box); therefore, the four dihedral atoms are CG3C51- CG2D1-CG2D2-HGA5.

Supplementary Table 1: Summary of NMR experiments

Solid State MAS NMR						
**ID**	**Sample**	**Experiment**	**B_0 (T)**	**ω_r (kHz)**	**CC mixing time (ms)**	**T (± 1°C)**
1	U-^{13}C,^{15}N-CA$_{CTD}$-SP1/BVM/IP6	2D CORD	20.0	14	10	4
		2D CORD	20.0	14	25	4
		2D CORD	20.0	14	50	4
		2D CORD	20.0	14	100	4
		2D CORD	20.0	14	200	4
		2D CORD	20.0	14	10	-10
		2D CORD	20.0	14	25	-10
		2D CORD	20.0	14	100	-10
		2D CORD	20.0	14	200	-10
		2D NCACX	20.0	14	50	4
		2D NCACX	20.0	14	50	-10
		2D CORD	14.1	14	25	4
		2D CORD	14.1	14	100	4
		2D CORD	14.1	14	250	4
		2D CORD	14.1	14	500	4
		2D INADEQUATE	14.1	14		4
		2D PAIN CP	14.1	14		4
2	U-^{13}C,^{15}N-CA$_{CTD}$-SP1/IP6	2D CORD	20.0	14	50	4
		2D CORD	20.0	14	100	4
		2D NCACX	20.0	14	50	4
		2D NCACX	20.0	14	50	-10
		3D NCOCX	20.0	14	25	4
		2D CORD	14.1	14	25	4
		2D CORD	14.1	14	50	4
		2D NCACX	14.1	14	25	4
		2D NCOCX	14.1	14	25	4
		3D NCACX	14.1	14	25	4
		2D NCACX	17.6	15	25	-79
		2D NCACX	17.6	15	25	-37
		2D NCOCX	17.6	15	25	-79
3	U-^{13}C,^{15}N-CA$_{CTD}$-SP1/BVM/SO_4	2D CORD	20.0	14	10	4
		2D CORD	20.0	14	50	4
		2D CORD	20.0	14	100	4
		2D CORD	20.0	14	200	4
		2D CORD	20.0	14	10	-10
		2D CORD	20.0	14	100	-10
		2D NCACX	20.0	14	50	4
		2D NCACX	20.0	14	50	-10

		2D INADEQUATE	20.0	14		4
		2D CORD	14.1	14	10	4
		2D CORD	14.1	14	25	4
		2D NCACX	14.1	14	25	4
		2D NCACX	14.1	14	50	4
		2D NCOCX	14.1	14	50	4
		3D NCACX	14.1	14	25	4
		3D NCOCX	14.1	14	25	4
		3D CONCA	14.1	14		4
4	U-^{13}C,^{15}N-CA$_{CTD}$-SP1/SO$_4$	2D CORD	20.0	14	50	4
		2D NCA	20.0	14		4
		2D NCACX	20.0	14	50	4
		2D CORD	14.1	14	25	4
		2D NCACX	14.1	14	25	4
		2D NCOCX	14.1	14	25	4
		2D NCOCX	14.1	14	50	4
		3D NCACX	14.1	14	25	4
		2D INADEQUATE	14.1	14		4
5	U-^{13}C,^{15}N,^{2}H-CA$_{CTD}$-SP1/BVM/IP6	1D dREDOR	20.0	40		4
		1D ^{31}P Direct	20.0	40		4
		1D ^{31}P CP	20.0	40		4
		2D CORD	20.0	14		-5
		2D HC CP HETCOR	20.0	40		4
		2D dREDOR-HETCOR	20.0	40		4
		2D NH HETCOR	20.0	40		4
		2D (H)PH HETCOR	20.0	40		4
6	U-^{13}C,^{15}N,^{2}H-CA$_{CTD}$-SP1/IP6 (Buffer A)	1D dREDOR	20.0	40		4
		1D ^{31}P Direct	20.0	40		4
		1D ^{31}P CP	20.0	40		4
		2D CORD	20.0	14		-5
		2D HC CP HETCOR	20.0	40		4
		2D dREDOR-HETCOR	20.0	40		4
		2D HC CP HETCOR	20.0	40		4
7	U-^{13}C,^{15}N,^{2}H-CA$_{CTD}$-SP1/IP6 (Buffer B)	2D CH HETCOR	20.0	40		4
		2D NH HETCOR	20.0	40		4
8	U-^{13}C,^{15}N,^{2}H-CA$_{CTD}$-SP1-V7A/BVM/IP6	1D ^{31}P Direct	20.0	40		4
		1D ^{31}P CP	20.0	40		4
		2D CORD	20.0	14		-5
		2D HC CP HETCOR	20.0	40		4
		2D (H)PH HETCOR	20.0	40		4
9	U-^{13}C,^{15}N,^{2}H-CA$_{CTD}$-SP1-V7A/IP6	1D ^{31}P Direct	20.0	40		4
		1D ^{31}P CP	20.0	40		4
		2D CORD	20.0	14		-5

		2D HC CP HETCOR	20.0	40		4
10	U-^{13}C,^{15}N,^{2}H-CA$_{CTD}$-SP1-A1V/BVM/IP6	1D ^{31}P Direct	20.0	40		4
		1D ^{31}P CP	20.0	40		4
		2D CORD	20.0	14		-5
		2D HC CP HETCOR	20.0	40		4
11	U-^{13}C,^{15}N,^{2}H-CA$_{CTD}$-SP1-A1V/IP6	1D ^{31}P Direct	20.0	40		4
		1D ^{31}P CP	20.0	40		4
		2D CORD	20.0	14		-5
		2D HC CP HETCOR	20.0	40		4
12	Powder IP6	2D (H)PH HETCOR	20.0	60		4
**Solution NMR**						
1	BVM/DMSO-D$_6$	1D ^{13}C	14.1			
		1D ^{1}H	14.1			
2	IP6/D$_2$O	1D ^{1}H	14.1			
		1D ^{31}P	14.1			

Supplementary Table 2: CGenFF and QM-modified dihedral angle parameters**.**

Fragment 3's Dihedral Angle Parameter				CGenFF			Penalty	QM-Modified				
				k	n	δ		k	n	δ	Δk	Δn
CG2D1	CG3C51	CG3C52	CG3C52	0.14	3	0	29	0.664	1	180	0.524	2
CG2D1	CG3C51	CG3C52	HGA2	0.14	3	0	29	0.445	3	0	0.305	0
CG2D1	CG3C51	CG3RC1	CG311	0.5	2	180	65.5	0.328	2	0	0.172	0
CG2D1	CG3C51	CG3RC1	CG3RC1	0.15	3	0	47	0.074	3	180	0.076	0
CG2D1	CG3C51	CG3RC1	HGA1	0.15	3	0	47	1.665	3	180	1.515	0
CG2D2	CG2D1	CG3C51	CG3C52	0.5<br>1.3	1<br>3	180<br>180	95	0.509	1	0	0.791	2
CG2D2	CG2D1	CG3C51	CG3RC1	0.5<br>1.3	1<br>3	180<br>180	98.8	0.509	1	0	0.791	2
CG2D2	CG2D1	CG3C51	HGA1	0.12	3	0	65	0.29	3	0	0.17	0
CG321	CG321	CG3RC1	CG2O3	0.8	4	180	74	1.473	1	180	0.673	3
HGA2	CG321	CG3RC1	CG2O3	0	3	0	74	0.976	1	180	0.976	2
CG2O3	CG3RC1	CG3RC1	CG311	4	3	0	75	1.99	3	0	2.01	0
CG2O3	CG3RC1	CG3RC1	CG3C51	0.15	3	0	52.4	0.252	3	180	0.102	0
CG2O3	CG3RC1	CG3RC1	HGA1	0.15	3	0	52	1.112	3	180	0.962	0
CG331	CG2D1	CG3C51	HGA1	0.19	3	0	65	0.666	1	180	0.476	2
OG2D2	CG2O3	CG3RC1	CG321	0.16	3	0	47	0.037	1	0	0.123	2
CG331	CG2D1	CG3C51	CG3C52	0.19	3	0	95	0.792	3	0	0.602	0
CG3C51	CG2D1	CG2D2	HGA5	5.2	2	180	10	5.2	2	180	0	0
CG3C51	CG2D1	CG331	HGA3	0.16	3	0	10	0.36	3	0	0.2	0
CG3C52	CG3C52	CG3RC1	CG2O3	0.14	3	0	16	0.524	3	0	0.384	0
OG2D2	CG2O3	CG3RC1	CG3C52	0.16	3	0	16	0.907	1	180	0.747	2
CG331	CG2D1	CG3C51	CG3RC1	0.19	3	0	98.8	0.692	1	180	0.502	2
HGA2	CG3C52	CG3RC1	CG2O3	0.14	3	0	16	1.01	1	180	0.87	2
OG2D2	CG2O3	CG3RC1	CG3RC1	0.16	3	0	57.1	0.761<br>1.244	2<br>3	0<br>0	1.084	0

Fragment 1's Dihedral Angle Parameter				CGenFF			Penalty	QM-Modified				
				k	n	δ		k	n	δ	Δk	Δn
CG2O3	CG301	CG321	CG2O2	0.2	3	0	10.5	0.997<br>0.115	1<br>3	180<br>180	0.085	0
CG311	CG301	CG311	OG302	0.2	3	180	12.6	0.31	1	180	0.110	2
CG331	CG301	CG311	OG302	0.2	3	0	12	0.362	1	0	0.162	2
CG331	CG301	CG321	CG2O2	0.2	3	0	12	0.187<br>0.997	3<br>1	180<br>0	0.013	0

The atom type in the first column can be identified in Supplementary Fig. 17. The penalty column represents the penalty score generated by CGenFF, which ranges from 12.0 to 98.8 and indicates these parameters

were required to be modified before involving in MD simulation. The last two columns represent the difference between the k and n values between CGenFF and QM-modified parameters. These values specify that the differences between CGenFF and QM-modified parameters are as low as <1.08, and therefore the QM-modified parameters have not significantly changed from CGenFF derived parameters. The highlighted columns demonstrate the modified parameters. Only parameters spotlighted in red in Supplementary Fig. 16a are presented in this table, and we accepted the rest parameters as CGenFF derived them.

Supplementary Table 3: Bond length, bond angle, and partial charges of BVM from CGenFF and QM-modified parameters**.**

Fragment 3's Bond Angle Parameter	CGenFF		Penalty	QM-Modified	
	k	θ_0		k	θ_0
CG2D2 CG2D1 CG3C51	48.0	126.0	10.0	97.710	125.4
CG331 CG2D1 CG3C51	48.0	123.5	10.0	81.76	114.19
CG2D1 CG3C51 CG3C52	52.0	112.0	29.0	90.70	110.77
CG2D1 CG3C51 CG3RC1	52.0	112.3	30.1	100.25	118.12
CG2D1 CG3C51 HGA1	50.0	112.0	29.0	61.2	108.23
CG2O3 CG3RC1 CG321	50.0	106.0	65.5	132.11	109.4
CG2O3 CG3RC1 CG3C52	52.0	112.3	16.0	107.57	106.73
CG2O3 CG3RC1 CG3RC1	70.0	113	16.0	124.41	115.77
**Fragment 3's Bond Length Parameter**	**CGenFF**		**Penalty**	**QM-Modified**	
	k	b_0		k	b_0
CG3RC1 CG2O3	250.0	1.49	16.0	190.310	1.579
CG3C51 CG2D1	365.0	1.502	65.0	258.150	1.523
**Fragment 1's Partial charges**	**CGenFF**		**Penalty**	**QM-Modified**	
	Partial charge			Partial charge	
CG2O2	0.902		11.5	0.802	
CG311	0.156		13.2	0.206	
CG301	0.018		13.3	0.068	

The atom type in the first column can be spotted by Supplementary Fig. 17. Only parameters spotlighted in red in Supplementary Fig. 16a are presented in this table, and we accepted the rest parameters as CGenFF derived them.

Supplementary Note 1: UCSF Chimera script for batch docking.

```
from chimera import runCommand as run, openModels
import re
import sys
import os

# Example of how to run this script:
# /Applications/Chimera.app/Contents/MacOS/chimera --nogui --script dock_chimera.py

# Load in pdb and density for docking
# Must have _ after number for strucIDs, otherwise just name it manually
in_pdb = "refine1_853.pdb"
stucIDs = re.search("\\d+_", in_pdb, re.M).group(0)

run('open ./54IT_noBVM_HOH_molmap_8A_resid148to238.mrc')
run(str("open ") + str(in_pdb))
# Set step level and make density a surface
run('volume #0 step 1')
run('volume #0 level 0.0063 style surface')
# Set this number high so no slots/fits missed
strucs_in_tube = "350"

# Set indicies and prepare for docking
map1_id = 1
map2_id = 0
# Set number of translations and rotations cross-correlation values (optional)
search = 250000
# Provide resolution. Script will run fine if this is approximate, the cross-
correlation
# values will be off but the values still will reveal the fits.
res = 8

# Execute the docking
from chimera import openModels as om, selection
m1 = om.list(id = map1_id)[0]
m2 = om.list(id = map2_id)[0]
s1 = selection.ItemizedSelection([m1])
from FitMap.fitcmd import fitmap
fit_list = fitmap(s1, m2, search = search, resolution = res, listFits = False)

# Access cross-corrleation value for each docked structure
print '%d fits' % len(fit_list)
import Matrix
corrs = []
for index, fit in enumerate(fit_list):
    if int(index) <= int(strucs_in_tube):
       print 'correlation =', fit.correlation()
    corrs.append(fit.correlation())
    if int(index) <= int(strucs_in_tube):
       fit.place_copies()

# Save pdb coordinates of fit with cross-correlation in the file-name
if not os.path.exists("Docked"):
       os.makedirs("Docked")
for index, fit in enumerate(fit_list):
       if int(index) <= int(strucs_in_tube):
              struc_ind = float(index) + 2
              print int(index), struc_ind
              outname = "Docked/docked_StrucNum_" + str(int(struc_ind)) + "_Corr_"
+ str(round(fit.correlation(), 4)) + ".pdb"
              print outname
              run(str("write ") + str(struc_ind) + str(" ") + outname)
```
