# Supplementary material for: Structural basis of HIV-1 maturation inhibitor binding and activity
Source: Nat Commun. 2023 Mar 4;14:1237. doi: 10.1038/s41467-023-36569-y (PMC9985623; doi:10.1038/s41467-023-36569-y)
Supplement: Supplementary file 2 — Description of Additional Supplementary File [file 41467_2023_36569_MOESM2_ESM.docx]

Description of Additional Supplementary File

File Name: Supplementary Data 1

Description: MAS NMR chemical shifts of CACTD-SP1/BVM/IP6 crystalline array
