# Supplementary material for: Structural basis of HIV-1 maturation inhibitor binding and activity
Source: Nat Commun. 2023 Mar 4;14:1237. doi: 10.1038/s41467-023-36569-y (PMC9985623; doi:10.1038/s41467-023-36569-y)
Supplement: Supplementary file 4 — Reporting Summary [file 41467_2023_36569_MOESM4_ESM.pdf]

nature portfolio

Corresponding author(s): Tatyana Polenova, Angela M. Gronenborn, Barbie K. Ganser-Pornillos, Owen Pornillos

Last updated by author(s): Dec 14, 2022

Reporting Summary

Nature Portfolio wishes to improve the reproducibility of the work that we publish. This form provides structure for consistency and transparency in reporting. For further information on Nature Portfolio policies, see our Editorial Policies and the Editorial Policy Checklist.

Statistics

For all statistical analyses, confirm that the following items are present in the figure legend, table legend, main text, or Methods section.

n/a	Confirmed	
☐	☒	The exact sample size (*n*) for each experimental group/condition, given as a discrete number and unit of measurement
☒	☐	A statement on whether measurements were taken from distinct samples or whether the same sample was measured repeatedly
☒	☐	The statistical test(s) used AND whether they are one- or two-sided<br>*Only common tests should be described solely by name; describe more complex techniques in the Methods section.*
☒	☐	A description of all covariates tested
☒	☐	A description of any assumptions or corrections, such as tests of normality and adjustment for multiple comparisons
☐	☒	A full description of the statistical parameters including central tendency (e.g. means) or other basic estimates (e.g. regression coefficient) AND variation (e.g. standard deviation) or associated estimates of uncertainty (e.g. confidence intervals)
☒	☐	For null hypothesis testing, the test statistic (e.g. *F*, *t*, *r*) with confidence intervals, effect sizes, degrees of freedom and *P* value noted<br>*Give P values as exact values whenever suitable.*
☒	☐	For Bayesian analysis, information on the choice of priors and Markov chain Monte Carlo settings
☒	☐	For hierarchical and complex designs, identification of the appropriate level for tests and full reporting of outcomes
☒	☐	Estimates of effect sizes (e.g. Cohen's *d*, Pearson's *r*), indicating how they were calculated

Our web collection on statistics for biologists contains articles on many of the points above.

Software and code

Policy information about availability of computer code

Data collection	NMR data were collected using Bruker TopSpin version 3.5 and 4.0, a standard commercial program.
Data analysis	NMR data were processed with Bruker TopSpin (version 4.1.3) and NMRPipe.<br>NMR spectra were Visualized and analyzed using NMRFAM-SPARKY (version 3.115) and CcpNmr Analysis (version 2.4). Torsion angle restraints were generated from experimental NMR data using TALOS-N web server (http://spin.niddk.nih.gov/bax/nmrserver/talosn).<br>Force field parameters of bevirimat (BVM) and inositol hexakisphosphate (IP6) were derived using CGENFF, GAUSSIAN16, and Force Field ToolkitM2.1 in VMD1.9.4.<br>MAS NMR structures of CACTD-SP1/BVM/IP6 and CACTD-SP1/IP6 were calculated and refined using X-PLOR NIH (version 2.53).<br>Restraint tallying and format conversions were carried out with in-house Python 2.7 scripts.<br>Structure ensembles were rendered for visualization in PyMOL 2.3.2 using in-house shell/bash scripts for batch rendering.<br>RMSD values were calculated using routines in the Xplor-NIH (version 2.51).<br>Secondary structure elements were classified according to TALOS-N.

For manuscripts utilizing custom algorithms or software that are central to the research but not yet described in published literature, software must be made available to editors and reviewers. We strongly encourage code deposition in a community repository (e.g. GitHub). See the Nature Portfolio guidelines for submitting code & software for further information.

Data

Policy information about availability of data

All manuscripts must include a data availability statement. This statement should provide the following information, where applicable:

- Accession codes, unique identifiers, or web links for publicly available datasets
- A description of any restrictions on data availability
- For clinical datasets or third party data, please ensure that the statement adheres to our policy

The MAS NMR atomic structure coordinates of single hexamer of CACTD-SP1/BVM/IP6 and CACTD-SP1/IP6 have been deposited in the Protein Data Bank under accession code 7R7P and 7R7Q respectively. MAS NMR chemical shifts of CACTD-SP1/BVM/IP6 and CACTD-SP1/IP6 have been deposited in the Biological Magnetic Resonance Data Bank under accession codes 30929 and 30930. The envelope of hexamer of hexamer of CACTD-SP1 used in this study is available in the Protein Data Bank under accession code 5I4T [http:doi.org/10.2210/pdb5I4T/pdb]. The initial coordinates of IP6 structure used in this study is available in the Protein Data Bank under accession code 6BHR [http:doi.org/10.2210/pdb6BHR/pdb]. Source data for viral infectivity assays are provided in this paper. Other data supporting the findings of study, for example, scripts for structure calculations, analysis of calculation results and structure visualizations, are available from the corresponding authors upon request.

Field-specific reporting

Please select the one below that is the best fit for your research. If you are not sure, read the appropriate sections before making your selection.

☒ Life sciences ☐ Behavioural & social sciences ☐ Ecological, evolutionary & environmental sciences

For a reference copy of the document with all sections, see nature.com/documents/nr-reporting-summary-flat.pdf

Life sciences study design

All studies must disclose on these points even when the disclosure is negative.

Sample size	All samples are described in the methods. The sample sizes are standard for the in vitro assays performed in the study.
Data exclusions	No data were excluded from the analyses.
Replication	Multiple samples have been measured by solid-state NMR, as described in the text, with consistent results. The measurements were replicated at least three times. All replication were successful.
Randomization	Randomization was not relevant to the study, as no human or animal subjects were studied, and the collected physical data were quantitative and did not require subjective interpretations.
Blinding	Blinding was not relevant to this study, as no subjective allocation was involved, and the results were quantitative and analyzed without subjective manual scoring.

Reporting for specific materials, systems and methods

We require information from authors about some types of materials, experimental systems and methods used in many studies. Here, indicate whether each material, system or method listed is relevant to your study. If you are not sure if a list item applies to your research, read the appropriate section before selecting a response.

Materials & experimental systems

n/a	Involved in the study	
☒	☐	Antibodies
☐	☒	Eukaryotic cell lines
☒	☐	Palaeontology and archaeology
☒	☐	Animals and other organisms
☒	☐	Human research participants
☒	☐	Clinical data
☒	☐	Dual use research of concern

Methods

n/a	Involved in the study	
☒	☐	ChIP-seq
☒	☐	Flow cytometry
☒	☐	MRI-based neuroimaging

Eukaryotic cell lines

Policy information about cell lines

Cell line source(s)	TZM-bl indicator cell line, HEK 293T cells (ATCC, Cat# CRL-3216)
Authentication	None of the cell lines used were authenticated.
Mycoplasma contamination	All cell lines tested negative for mycoplasma contamination.
Commonly misidentified lines (See ICLAC register)	None
